# Neodymium magnetic field meets nanocatalysis: a sustainable route to novel azines and condensed heterocycles

**DOI:** 10.1038/s41598-026-51258-8

**Published:** 2026-05-21

**Authors:** H. A. Morsy, Ahmed H. Moustafa, Hassan A. El-Sayed, Mohamed G. Assy, Doaa A. Elsayed

**Affiliations:** 1 Higher Institution of Engineering & Modern Technology, Elmarg, Cairo, 13774 Egypt; 2 Department of Chemistry, Faculty of Science, Zagazig University, Zagazig, 44519 Egypt

**Keywords:** Neodymium magnetic, Static magnetic field, Nano particles, Green chemistry, Azines, Chemistry, Nanoscience and technology

## Abstract

A sustainable and effective approach for manufacturing heterocyclic compounds was established utilizing Fe₃O₄ nanoparticles in the presence of a neodymium static magnetic field (about 9000 G). Pyrimidine, benzimidazole, quinoxaline, and benzodiazepine derivatives were swiftly synthesized at ambient temperature with excellent efficiency; no reaction transpired in the absence of a magnetic field. TEM and VSM investigations validated nanoscale dimensions (9–50 nm) and robust magnetic characteristics, facilitating efficient catalysis and straightforward recovery. The produced compounds were validated using FT-IR, ^1^H NMR, ^13^C NMR, and elemental analysis. The technology markedly decreased reaction time, reduced energy consumption, eliminated hazardous chemicals, and offers a sustainable pathway for synthesizing physiologically relevant heterocycles.

**Supplementary Information:**

The online version contains supplementary material available at 10.1038/s41598-026-51258-8.

## Introduction

The advancement of eco-friendly, sustainable synthetic methods has become a primary goal in contemporary organic chemistry. The notion of green chemistry, articulated through the twelve principles established by Anastas and Warner, underscores the reduction of waste, atom economy, the utilization of cleaner solvents, energy efficiency, and the preference for catalytic over stoichiometric reagents. These concepts seek to minimize the environmental impact of chemical processes while ensuring high efficiency, selectivity, and practicality in organic synthesis^1–6^. As a result, considerable efforts have been devoted to developing more environmentally friendly synthetic methods that reduce reliance on toxic chemicals and enhance overall process sustainability, as evidenced by recent research**.**

Recent years have seen the development of several environmentally benign synthetic methods for organic transformations. These include methods such as electrochemical processes, visible-light photocatalysis, microwave-assisted synthesis, ultrasonic irradiation, and mechanochemical procedures like ball milling. Significant benefits in organic synthesis, such as increased selectivity, reduced solvent use, shorter reaction times, and higher yields, have been demonstrated using these techniques^7–13^.

Several environmentally benign synthetic methods have been established for organic transformations, including microwave-assisted synthesis, ultrasonic irradiation, visible-light photocatalysis, electrochemical procedures, and mechanochemical processes such as ball milling^14,15^. These techniques offer significant benefits in organic synthesis, including shorter reaction times, higher yields, reduced solvent use, and greater selectivity. Notwithstanding these advancements, the persistent quest for more efficient, scalable, and ecologically sustainable techniques remains a pivotal factor in synthetic chemistry^16–21^.

Nanotechnology has emerged as a powerful tool in green chemistry, offering innovative solutions for catalytic efficiency and process sustainability. In particular, nanocatalysts exhibit high surface area, enhanced reactivity, and excellent selectivity compared to bulk materials. Among them, magnetically recoverable nanocatalysts, especially Fe₃O₄-based systems, have gained significant attention due to their facile magnetic separation, recyclability, and operational simplicity^22–26^. These features make them highly attractive for sustainable organic transformations.

**Fig. 1:**
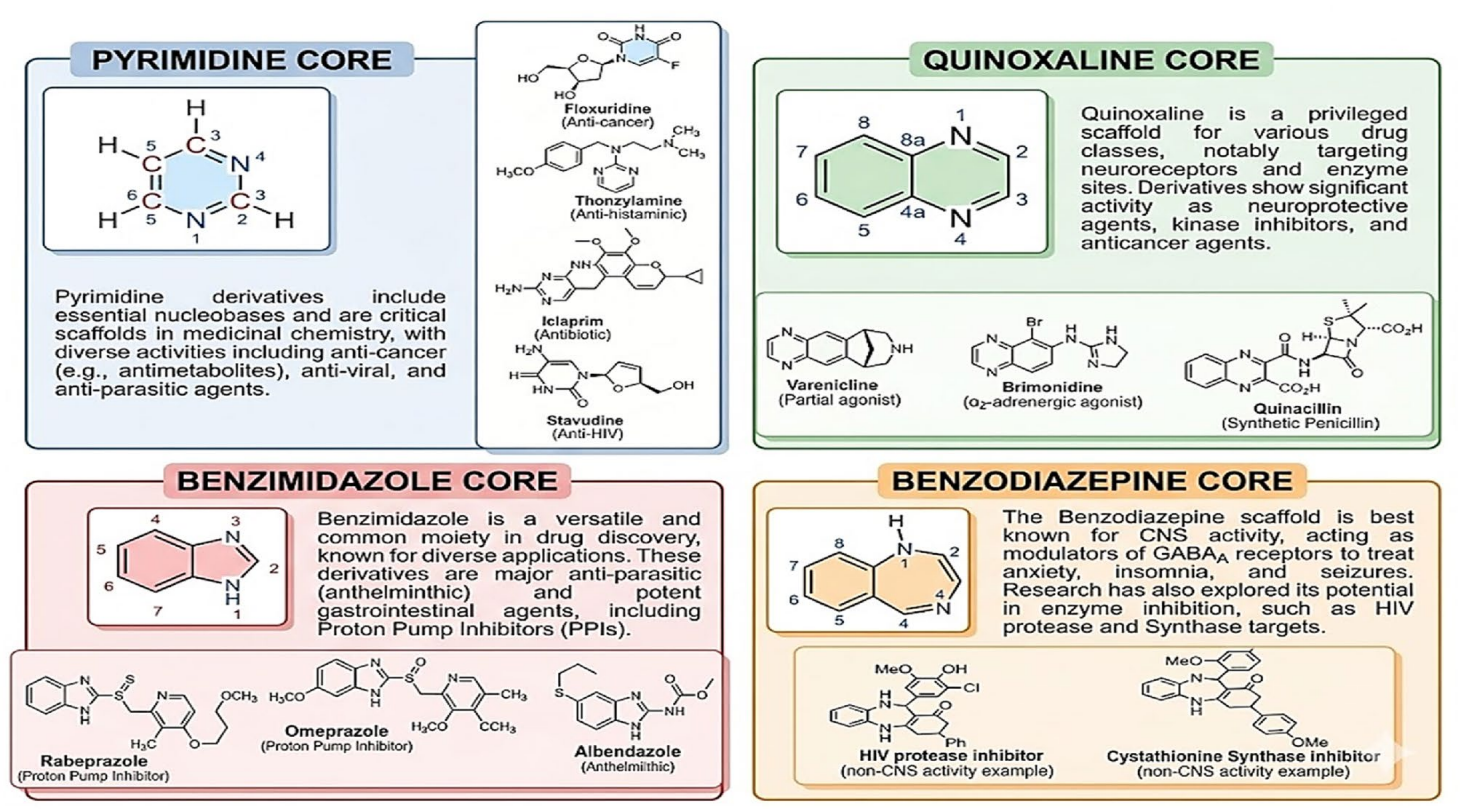
Structural representation and pharmacological significance of privileged heterocyclic scaffolds. The figure illustrates examples of clinically approved drugs and biologically active compounds containing pyrimidine, benzimidazole, quinoxaline, and benzodiazepine as core moieties.

Recent studies have revealed a diverse array of green organic transformations, encompassing reductive amination of carbonyl compounds, acylation reactions, multicomponent condensations, and heterocycle synthesis^27–34^, frequently conducted under solvent-free or mild conditions, yielding excellent results with minimal side reactions. Additionally, diverse advanced nanocatalysts, including silica-coated magnetic nanoparticles, molybdate-supported systems, and bio-derived or mixed-metal oxides, have demonstrated exceptional catalytic efficacy, operational ease, and substantial recyclability across numerous cycles without significant loss of activity. These advancements underscore the essential role of magnetic nanocatalysts in developing more environmentally friendly, efficient, and sustainable synthesis methods, highlighting their growing significance in contemporary organic chemistry^35–41^**.** Heterocyclic compounds constitute a crucial category of organic molecules in medical chemistry and pharmaceutical research. Over 90% of biologically active chemicals and commercial pharmaceuticals have at least one heterocyclic moiety within their molecular structure^42–45^. These heterocycles are not merely structural components; they are indispensable pharmacophores that modulate biological potency, refine pharmacokinetic profiles, and enhance binding affinities with specific molecular targets. Figure 1 summarizes key pharmacologically active heterocyclic scaffolds, including pyrimidine, benzimidazole, quinoxaline, and benzodiazepine, which form the structural basis of numerous therapeutic agents.

**Fig. 2:**
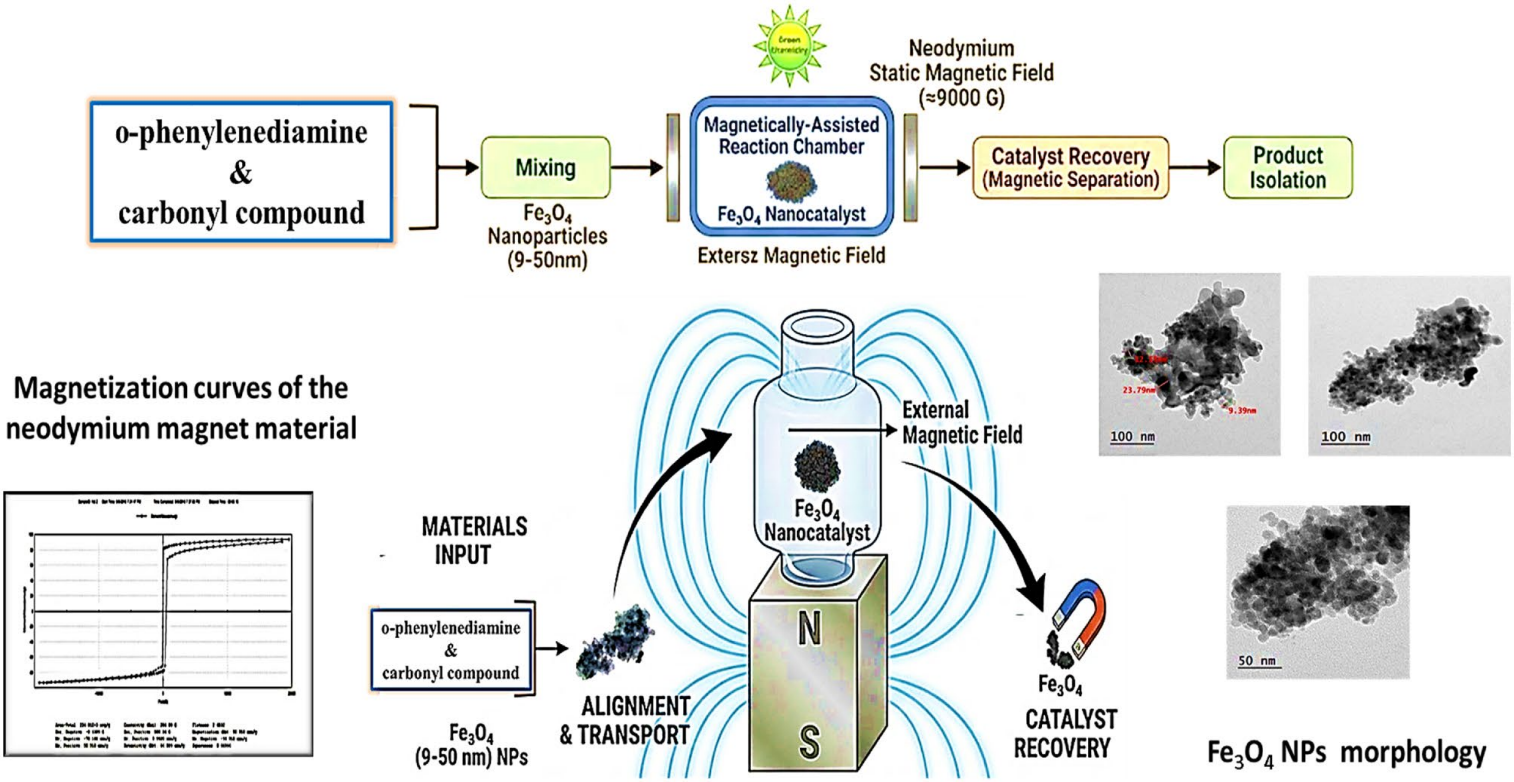
Diagram illustrating magnetically-assisted synthesis, catalysis, characterization, and recovery processes for sustainable green chemistry application.

To the best of our knowledge, the application of static external magnetic fields in combination with Fe₃O₄-based nanocatalysts for enhancing organic transformations, particularly heterocyclic synthesis, remains underexplored. In this context, the present work introduces a novel and sustainable catalytic strategy that integrates Fe₃O₄ nanoparticles with a neodymium static magnetic field to enhance reaction efficiency under mild, eco-friendly conditions. This magnetic-field-assisted approach offers improved reaction rates, reduced reaction time, facile catalyst recovery, and excellent recyclability. This study aims to evaluate the effectiveness of this hybrid system in the synthesis of biologically relevant heterocyclic compounds as a green and efficient alternative to conventional methods, Fig. 2.

## Result and discussion

### Magnetic characterization of a neodymium magnet by VSM analysis

**Fig. 3:**
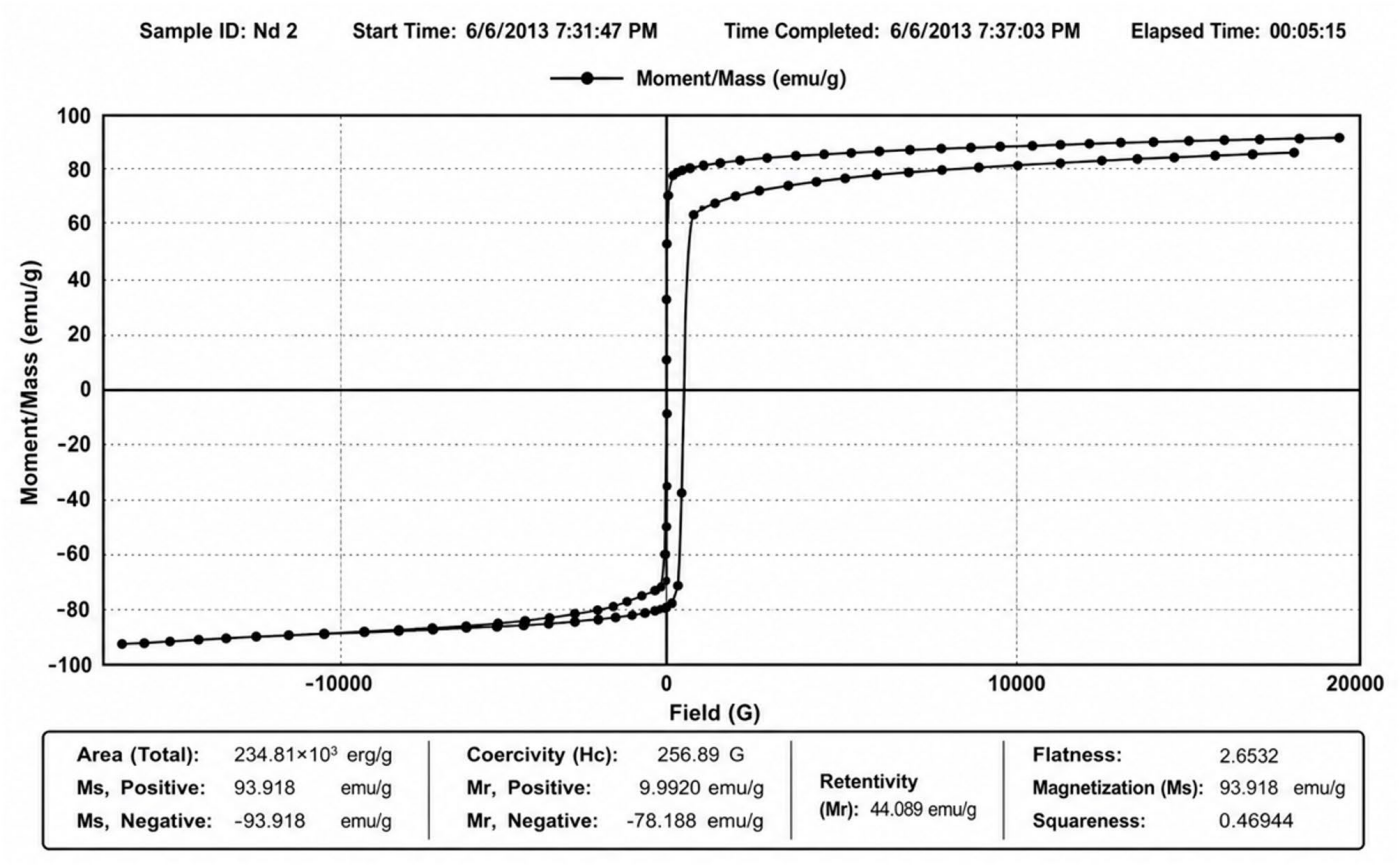
Magnetization curves of the neodymium magnet material.

The magnetic characteristics of the neodymium magnet were examined using the Vibrating Sample Magnetometer (VSM) method at ambient temperature (Fig. 3). The VSM analysis yields critical insights into the magnetic properties of materials by quantifying magnetization as a function of the applied magnetic field.

The acquired hysteresis loop demonstrated that the neodymium magnet had robust magnetic properties, with a magnetization value of 93.318 emu g⁻^1^. The elevated magnetization substantiates the superior magnetic properties of the neodymium material, making it highly suitable for applications requiring robust magnetic fields. Elevated magnetization indicates that the material exhibits favorable ferromagnetic characteristics.

The magnetic performance of nanoparticles is crucial in catalytic systems using magnetic nanoparticles, since robust magnets enhance the efficient magnetic separation and retrieval of catalysts from reaction mixtures. Consequently, the elevated magnetic value derived from the VSM study substantiates the prospective application of neodymium magnets in magnetically assisted catalytic processes and nanoparticle manipulation.

### Transmission electron microscopy (TEM) analysis of Fe_3_O_4_ nanoparticles

**Fig. 4:**
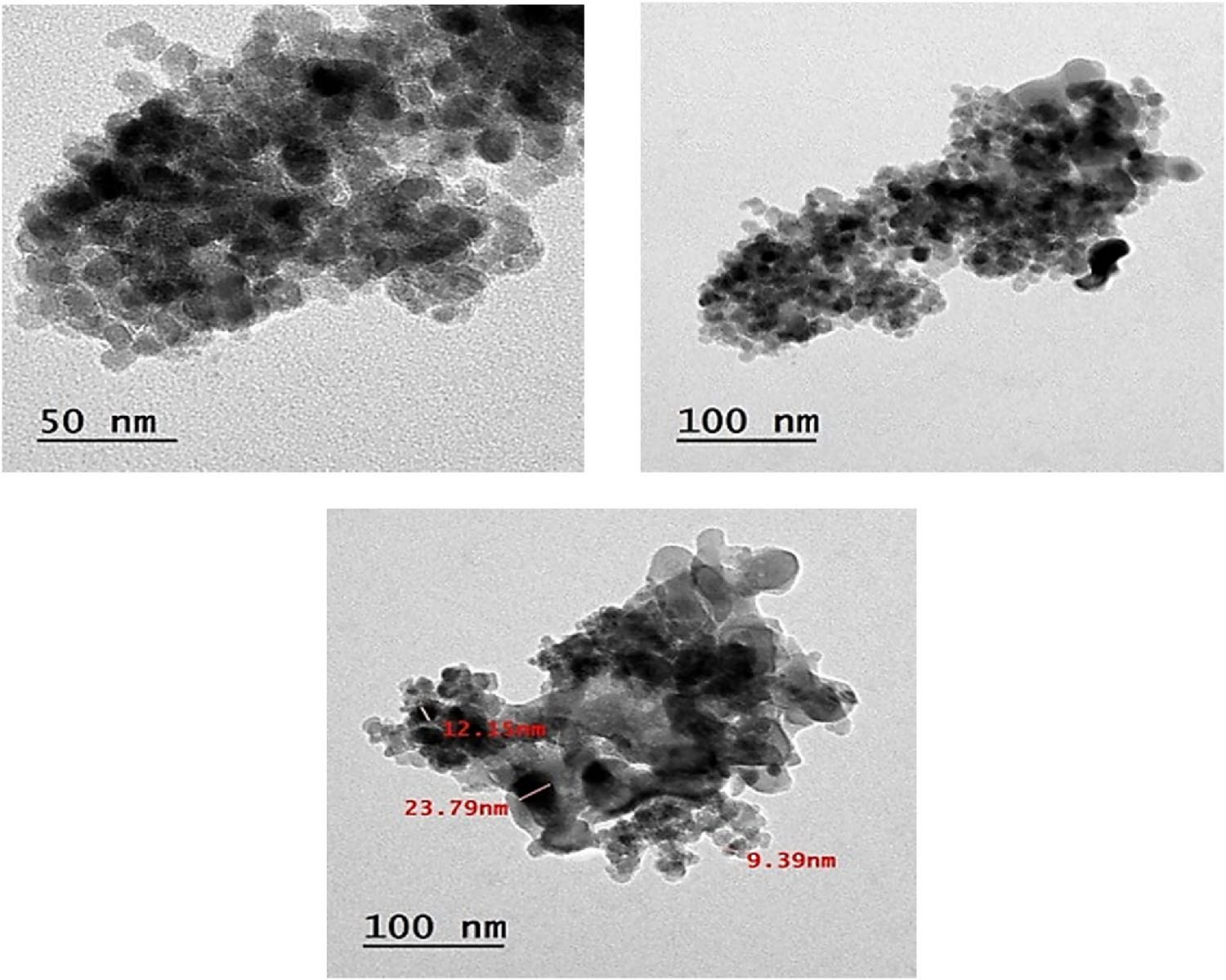
TEM analysis of Fe_3_O_4_ nanoparticles.

The shape and particle size distribution of the produced Fe_3_O_4_ nanoparticles were analyzed using Transmission Electron Microscopy (TEM). Transmission Electron Microscopy (TEM) is an advanced method that enables direct observation of nanoparticle architecture and yields precise data on their dimensions and morphology. Figure 4 illustrates that the TEM micrographs indicate the Fe_3_O_4_ nanoparticles have a rather consistent shape with well-defined nanoscale structure. The particles predominantly exhibit a spherical-to-nearly-spherical morphology and are fairly well distributed, although some aggregation may occur due to magnetic interactions among the particles. The particle size study reveals that the average nanoparticle size ranges from 9.39 to 50 nm, thereby confirming the effective synthesis of nanoscale magnetite particles. Nanoscale dimensions are especially beneficial for catalytic and magnetic applications, as reduced particle sizes yield an increased surface area, hence augmenting reactivity and contact with adjacent molecules. The observed size distribution and morphology indicate that the synthesis process employed in this work successfully produced Fe3O4 nanoparticles with suitable structural attributes for prospective applications in catalysis, environmental remediation, and magnetically recoverable systems.

### PXRD characterization of Fe₃O₄ NPs

**Fig. 5:**
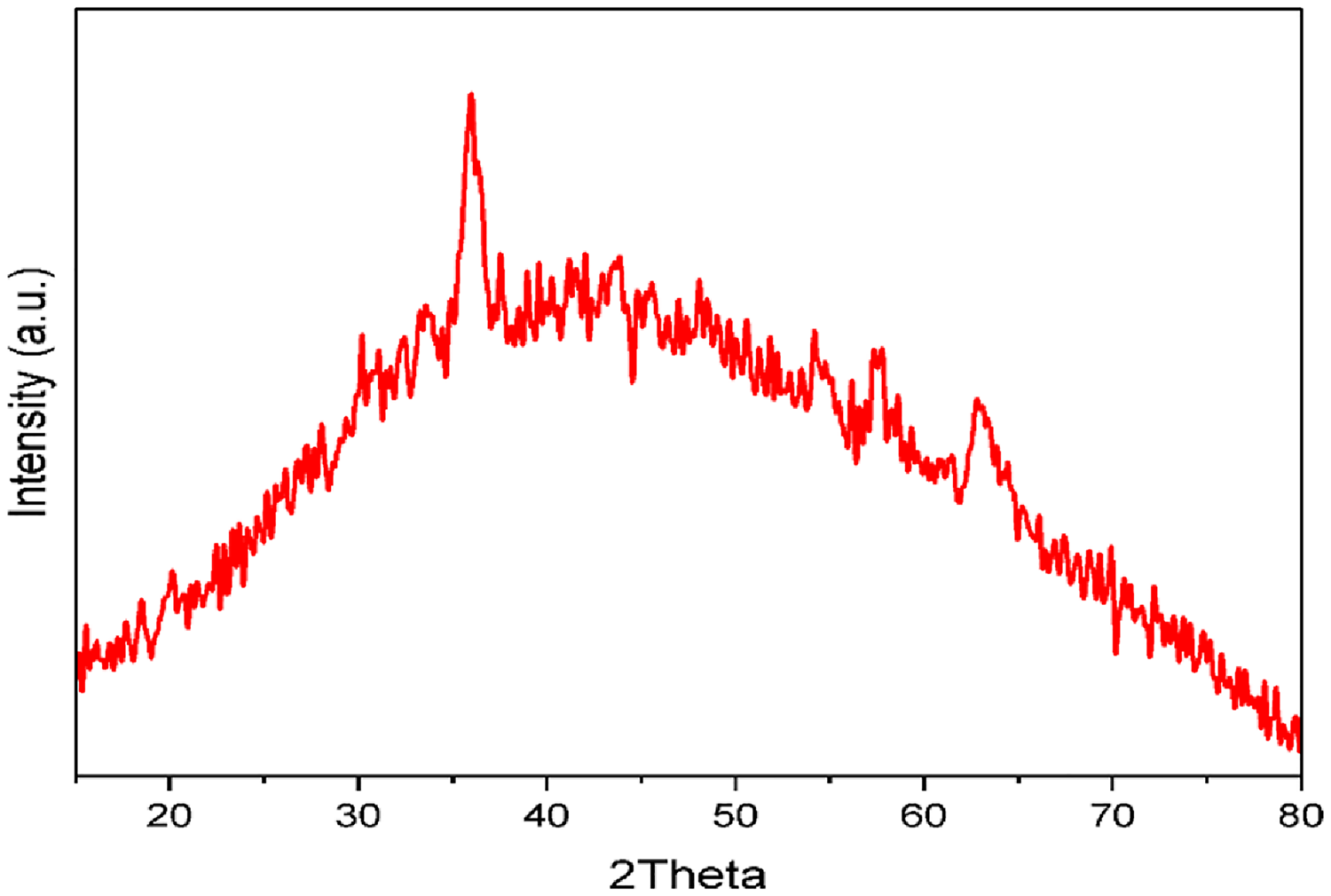
XRD pattern of Fe₃O₄ NPs.

The crystal structure and phase purity of the produced material were examined via X-ray diffraction (XRD). The acquired diffraction pattern displayed distinct characteristic peaks at 2θ values of roughly 18.36°, 30.21°, 35.99°, 37.22°, 43.25°, 47.36°, 53.67°, 57.21°, and 62.83°, which were correlated to the crystallographic planes (111), (220), (311), (222), (400), (331), (422), (511), and (440), respectively, Fig. 5. These observations align with the cubic inverse spinel configuration of magnetite (Fe₃O₄) characterized by the space group Fd-3 m. The diffraction pattern aligns perfectly with the standard crystallographic data from the Crystallography Open Database (COD card No. 7228110) and the standard reference pattern (JCPDS card No. 19–0629), thereby confirming the successful synthesis of Fe₃O₄. The prominent diffraction peak seen at 2θ = 35.99°, associated with the (311) plane, is indicative of magnetite, signifying the high crystallinity of the produced nanoparticles, and the lattice parameter was determined to be a = 8.36 Å, aligning with documented values for cubic Fe₃O₄^46,47^**.**

### Synthesis of heterocyclic derivatives in a static magnetic field

**Fig. 6:**
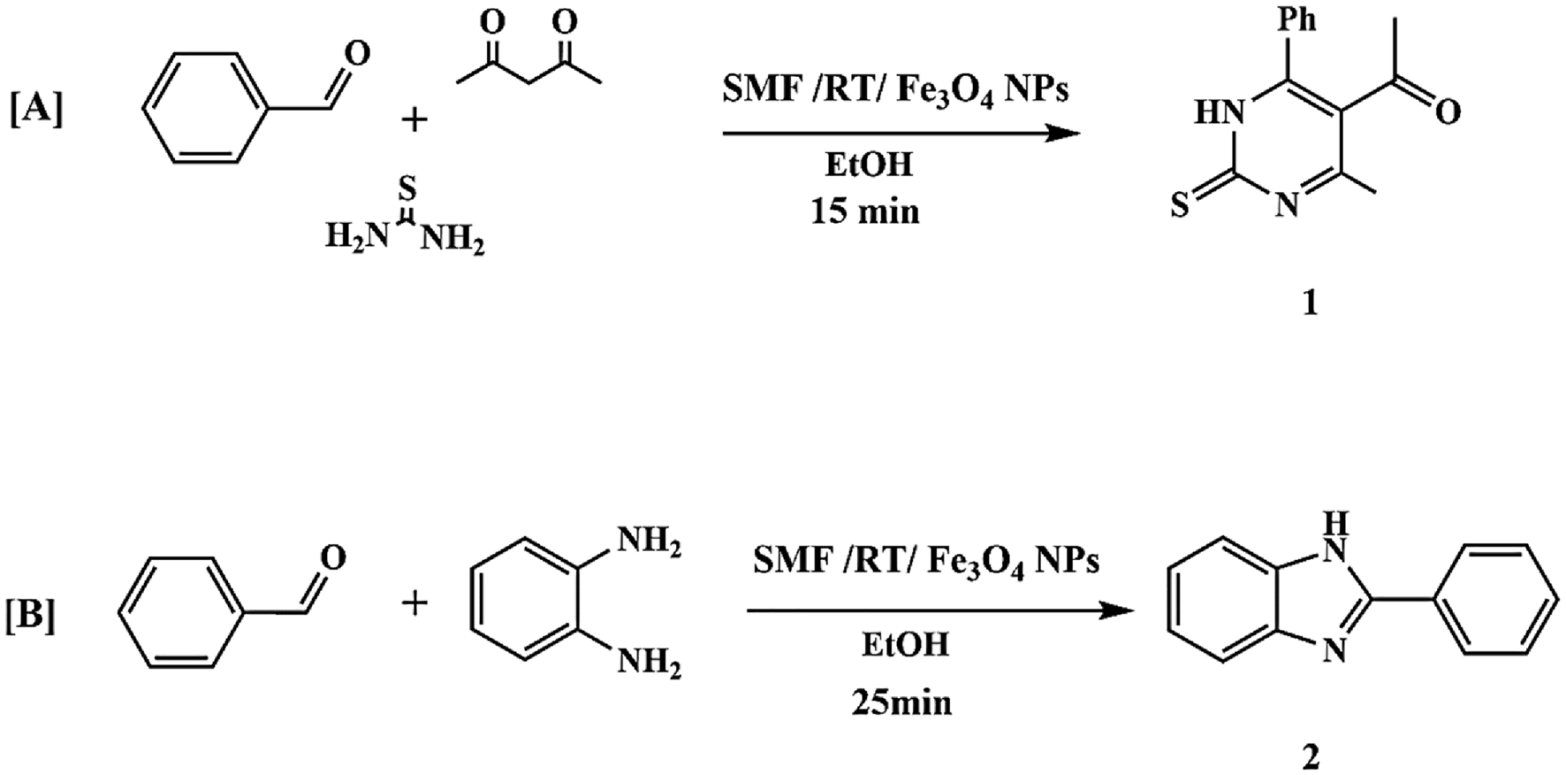
Synthesis of pyrimidine and benzimidazole.

This study successfully synthesized a series of heterocyclic derivatives **(1–8)** using a modified synthetic strategy that employed a static magnetic field (SMF) generated by a strong permanent neodymium magnet (≈9000 G) in conjunction with magnetite nanoparticles (Fe_3_O_4_ NPs) as a catalytic support. The comprehensive synthetic pathways are depicted in Figs. 6, 8, and 9. This study presents an ecologically benign way for synthesizing heterocyclic systems utilizing magnetic-field-assisted techniques, in contrast to previously described conventional procedures.

The reactions were conducted in absolute ethanol at ambient temperature utilizing Fe_3_O_4_ nanoparticles (1 mmol) as a heterogeneous catalyst. The use of an external magnetic field on the reaction mixtures markedly expedited the processes, facilitating the synthesis of the targeted heterocyclic compounds in 15 to 25 min.

Compound (1) was produced by a multicomponent condensation process involving benzaldehyde, acetylacetone, and thiourea, yielding the appropriate tetrahydropyrimidine derivative. Likewise, the condensation of o-phenylenediamine with benzaldehyde under the same circumstances produced 2-phenyl-1H-benzimidazole (2) with a yield of 34%, Fig. 6.

**Fig. 7:**
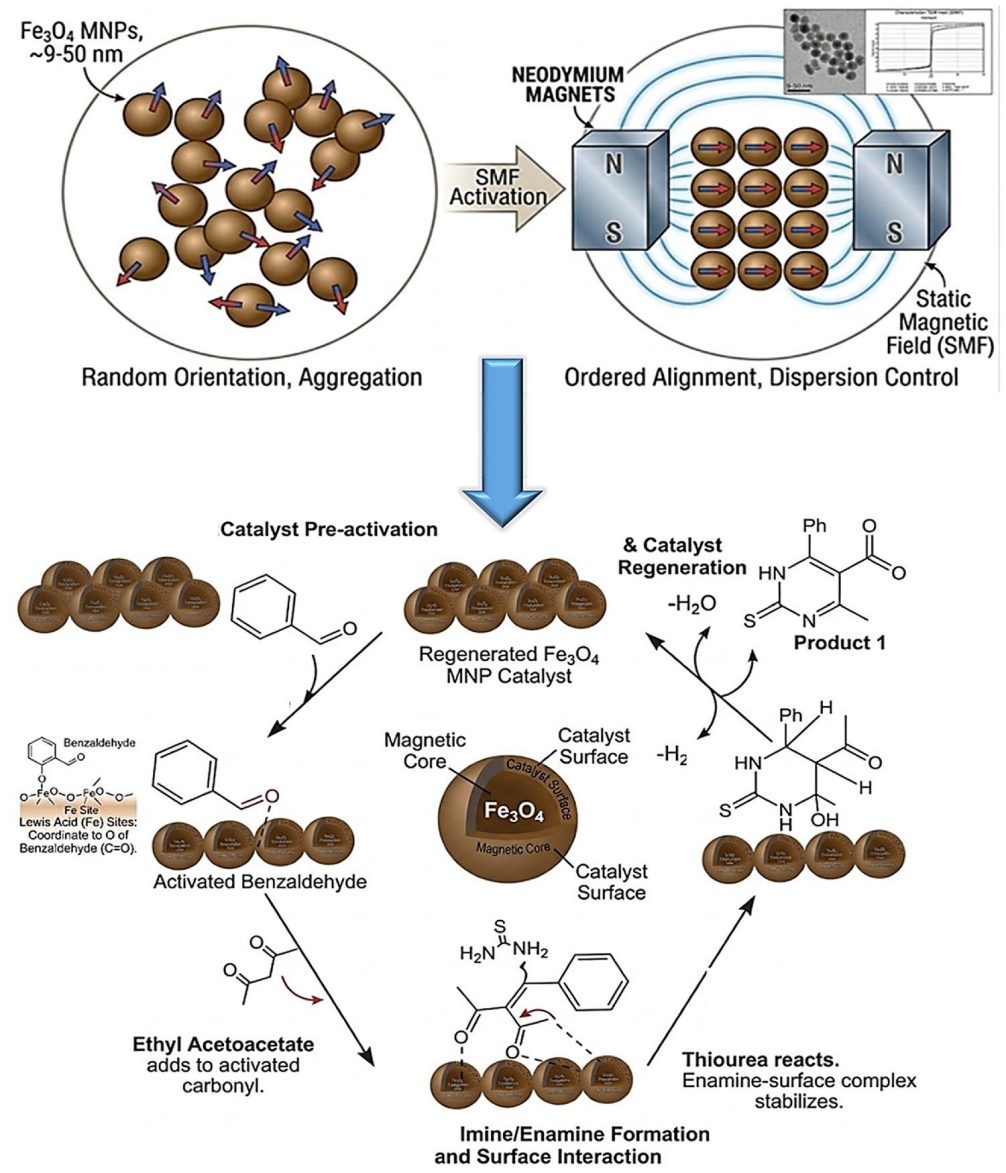
Proposed synergistic mechanism for the synthesis of pyrimidinethione derivatives over Fe_3_O_4_ MNPs.

The synergistic integration of Fe3O4 Magnetic nanoparticles (MNPs) and an external static magnetic field (SMF) significantly enhances catalytic efficiency in the synthesized multicomponent reaction. In the absence of an SMF, MNPs typically exhibit random orientation and inherent aggregation due to magnetic dipole–dipole interactions, which limits the accessible active surface area^48^. However, upon application of a 9000 G SMF, the nanoparticles undergo ordered, anisotropic alignment, promoting superior dispersion and preventing cluster formation^49^. This physical reorientation facilitates optimal exposure of the surface Fe sites, which act as potent Lewis acid centers^50^. The mechanism begins with coordination of the benzaldehyde carbonyl oxygen to the Fe sites, thereby inducing electrophilic activation. This is followed by a sequential Michael addition of ethyl acetoacetate, followed by a nucleophilic attack by thiourea. The stabilized imine/enamine-surface complex undergoes cyclization and dehydration, yielding the final dihydropyrimidinethione product. The paramagnetic nature of the Fe_3_O_4_core ensures rapid catalyst recovery via an external magnet, maintaining high recyclability without significant loss of structural integrity, Fig. 7.

**Fig. 8:**
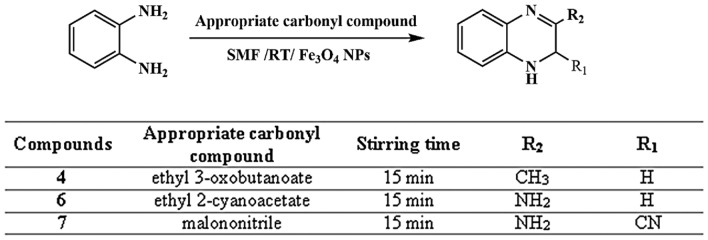
Synthesis of quinoxaline derivatives.

**Fig. 9:**
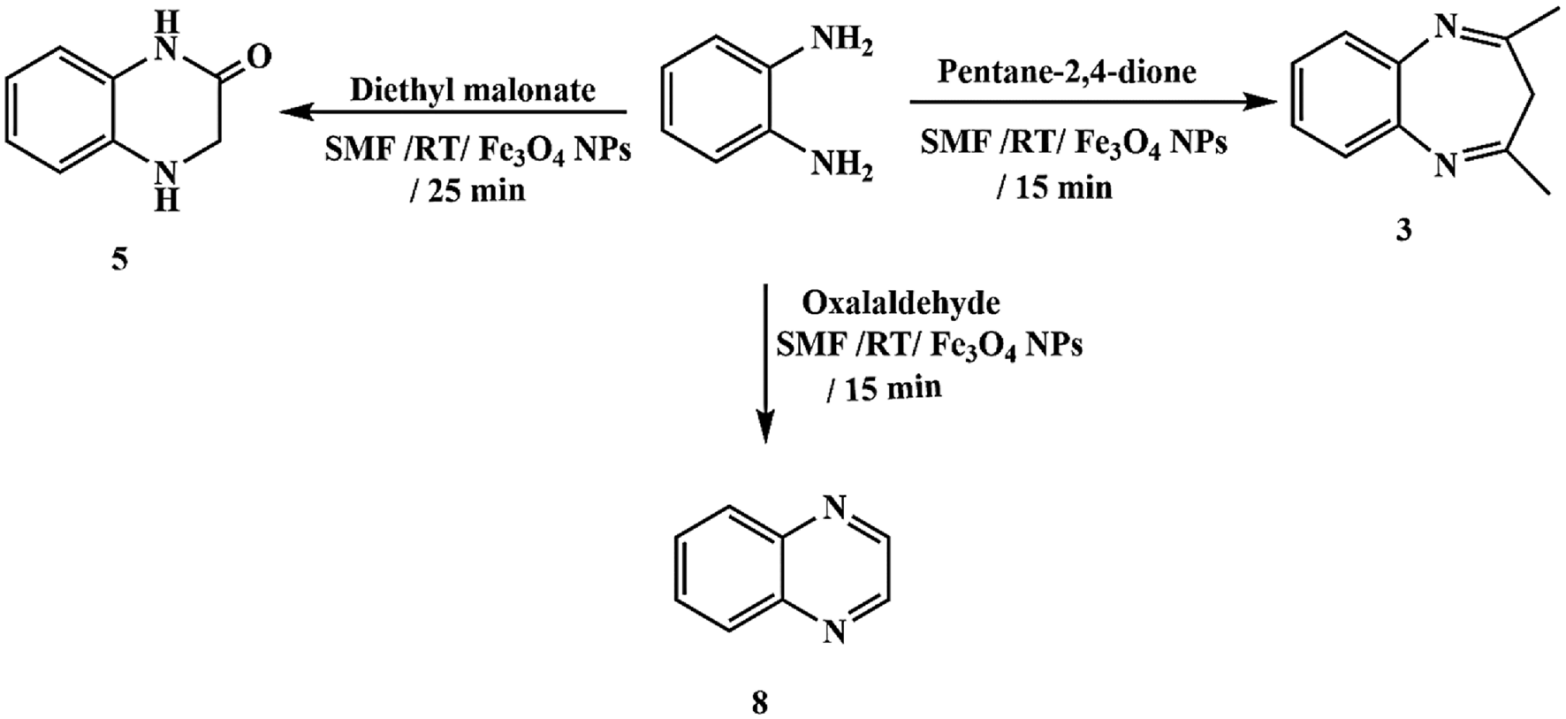
Synthesis of benzodiazepine and quinoxaline derivatives.

Moreover, the interaction of o-phenylenediamine with diverse dicarbonyl compounds, including acetylacetone, ethyl acetoacetate, diethyl malonate, ethyl cyanoacetate, malononitrile, and glyoxal, yielded multiple heterocyclic structures such as benzodiazepine, quinoxaline, and quinoxaline derivatives **(3–8)**. The reactions proceeded efficiently in the presence of the magnetic field, with reaction durations of 15 min, except for compound (5), which required 25 min to complete, Figs. 8 and 9.

### Structural analysis of the synthesized compounds

The structures of the synthesized heterocyclic compounds **(1–8)** were validated using a mix of spectroscopic methods, including FT-IR, ^1^H NMR, ^13^C NMR, and elemental analysis. The FT-IR spectra of the synthesized compounds **(1–8)** provided strong evidence of the successful formation of the desired heterocyclic frameworks by identifying their characteristic functional groups. The observed vibrational frequencies were in good agreement with previously reported values for structurally similar compounds, confirming the reliability of the assigned structures.

For compound (1), a broad absorption band observed at 3120 cm⁻^1^ was attributed to N–H stretching vibration. In addition, the characteristic band at 1257 cm⁻^1^ is assigned to C = S stretching, which is typical for thioxo-pyrimidine derivatives and consistent with reported spectral data for thiourea-derived heterocycles in the literature^51–53^. These findings strongly support the successful cyclocondensation leading to the formation of the target tetrahydropyrimidinethione scaffold.

For compounds (2–8), the FT-IR spectra exhibited characteristic absorption bands corresponding to the expected heterocyclic functionalities. In particular, bands appearing in the range of 1580–1650 cm⁻^1^ were assigned to C = N stretching vibrations, confirming the formation of imine-containing heterocycles such as benzimidazole, quinoxaline, and benzodiazepine derivatives. The broad absorption bands observed around 3160–3330 cm⁻^1^ were attributed to N–H and NH₂ stretching vibrations, further supporting the presence of nitrogen-rich heterocyclic systems^54–56^. Moreover, the appearance of a sharp absorption band at 2194 cm⁻^1^ in compound (7) is characteristic of the nitrile (C≡N) group, providing strong evidence for successful cyano-substitution and is in agreement with previously reported values for cyano-functionalized heterocycles^56^.

Additionally, the carbonyl stretching vibration observed at 1665 cm⁻^1^ in compound (5) confirms the presence of (C = O) functionality, which is consistent with quinoxalinone derivatives reported in the literature^55^. Overall, the FT-IR data collectively validate the successful synthesis of the target heterocyclic compounds, with all observed vibrational frequencies showing excellent agreement with previously reported literature values for similar systems ^57–60^.

The ^1^H NMR spectra corroborated the hypothesized structures. In compound (1), the singlet signal at δ 10.60 ppm corresponds to the NH proton, whereas the aromatic protons are seen as a multiplet around δ 7.31 ppm. The methyl protons were observed at δ 1.2 ppm and δ 4.21 ppm, consistent with the proposed tetrahydropyrimidine structure. The spectral data for compounds (2–8) displayed distinctive signals corresponding to aromatic protons, methylene groups, and heterocyclic NH protons. The elemental analysis results were in close agreement with the estimated values for the proposed molecular formulae, further validating the successful synthesis and purity of the identified compounds.

### Nature and physicochemical properties of the synthesized organic materials

The synthesized compounds (1–8) represent a structurally diverse class of nitrogen-containing heterocyclic organic materials, including thioxotetrahydropyrimidine, benzimidazole, benzodiazepine, and quinoxaline derivatives, which are widely recognized as privileged scaffolds in medicinal and synthetic organic chemistry due to their broad spectrum of biological and electronic properties^42,61,62^. These systems are characterized by conjugated aromatic frameworks and heteroatoms (N, O, S), which significantly influence their physicochemical stability, polarity, and reactivity^62,63^.

The incorporation of heteroatoms enhances hydrogen-bonding capability and electron delocalization within molecular frameworks, leading to increased structural rigidity and improved thermal stability. Compounds bearing carbonyl, thiocarbonyl, and cyano functionalities (e.g., compounds **1** and **7**) exhibit extended π-conjugation, which plays a key role in modulating their electronic distribution and potential interaction with biological targets^64^.

From a physicochemical perspective, the relatively high melting points observed (133–296 °C) reflect strong intermolecular interactions, such as π–π stacking and hydrogen-bonding networks, which are characteristic of fused heterocyclic systems. Moreover, the solubility behavior in polar aprotic solvents (e.g., DMSO) is consistent with the presence of polar functional groups, including NH, NH₂, C = O, and CN, which increase molecular polarity and intermolecular interactions.

Substituent effects further modulate the electronic properties of the synthesized compounds. Electron-donating groups, such as methyl and amino substituents, increase electron density on the heterocyclic core, whereas electron-withdrawing groups, such as cyano groups, decrease electron density and enhance electrophilic character, thereby influencing reactivity and potential binding affinity in biological systems^65,66^. Such tunable electronic properties are a key feature of heterocyclic compounds in drug design and molecular recognition processes.

The synthesized heterocyclic frameworks exhibit well-defined structural, thermal, and electronic properties, making them promising scaffolds for further computational, pharmacological, and materials science investigations^67^.

### Effect of solvent on reaction efficiency

To establish the optimal reaction conditions, solvent optimization was carried out under the same reaction conditions. Among the tested solvents, ethanol afforded the highest yield (≈34%), methanol gave a lower yield (25%), and water showed only trace product formation. The solvent-free condition (neat) was also less effective (≈12%). These results indicate that ethanol is the most suitable medium for this transformation (Table 1).

**Table 1:** Effect of solvent.

Entry	Solvent	Yield (%)
1	Ethanol	≈34
2	Methanol	25
3	Water	Traces
4	Neat	≈12

### Magnetic field consideration

A neodymium magnet (rectangular prism, 90 × 40 × 100 mm) was employed to generate a static magnetic field during the reaction. However, due to the relatively low Curie temperature of neodymium magnets, prolonged exposure to elevated temperatures may lead to irreversible loss of magnetization^68^. Therefore, moderate reaction conditions were adopted to preserve the magnet’s magnetic properties for repeated use in ongoing studies.

### Catalyst recycling and reusability

After completion of the reaction, a neodymium magnet (20 × 3 × 50 mm) was inserted into the reaction vessel to enable magnetic separation of the catalyst. The recovered catalyst was meticulously cleaned with absolute ethanol and subsequently dried under vacuum before reuse. The catalyst was effectively recycled over four successive cycles with no significant weight loss. The reusability of the magnetic catalyst was further evaluated over four consecutive reaction cycles under the optimized conditions. As shown in Table 2, the catalyst maintained nearly consistent catalytic activity, with only a slight decrease in yield from 34% in the first cycle to 32% in the fourth cycle. This minimal reduction confirms the catalyst’s excellent stability and recyclability under the reaction conditions applied. The use of magnetic nanocatalysts for facile recovery and reuse has been widely reported in the literature, demonstrating their advantages in green and sustainable chemistry^69–71^**.**

**Table 2:** Catalyst recycling.

Cycle	Yield (%)
1	34
2	34
3	33
4	32

### Discussion of the comparative study

The efficacy of the current synthetic process was assessed by comparing it with previously reported methodologies for the synthesis of analogous heterocyclic molecules (Table 3). The methodologies described in the literature typically require prolonged reaction times, ranging from 60 min to several hours, often under reflux, at elevated temperatures, or with intricate catalytic systems such as ionic liquids, metal complexes, or acid-supported catalysts.

**Table 3:** Comparison of reaction conditions for the synthesis of selected heterocyclic compounds using literature methods and the present SMF-assisted approach.

Compound	Reaction Conditions	Catalyst	Time	Melting Point	Ref
	EtOH / reflux	HCl	8 h	197–199 °C	^72^
	Neat / 100 °C	Cu(II)NDs@CFG	70 min	183–184 °C	^73^
	H₂O/EtOH (3:1), 70 °C	Xylose	70 min	292–294 °C	^74^
	MeOH / RT/stirring	Co(acac)₂	240 min	296.5–297.5 °C	^75^
	CH₃CN/H₂O, 55 °C	[MIMPs]⁺Cl⁻ / TEMPO / NaNO₂	300 min	292–293 °C	^76^
	100 °C	H₂SO₄–SiO₂	60 min	135 °C	^77^
	CH₃CN / r.t	Triethylamine	10 min	136–138 °C	^78^
	MeOH / r.t. / stirring	Co(acac)₂	240 min	29–31 °C	^75^
**Present Work (1–8)**	**EtOH / r.t. under SMF (9000 G)**	**Fe**_**3**_**O**_**4**_** NPs**	**15–25 min**	**Consistent with literature values**	**This work**

For instance, the synthesis of tetrahydropyrimidine derivatives (DHPM) documented in previous research required 8 h under reflux or 70 min at 100 °C using copper nanodot catalysts^72,73^. The synthesis of benzimidazole derivatives required reaction times ranging from 70 to 300 min, 72–74, depending on the catalyst and solvent system used. Moreover, the synthesis of quinoxaline derivatives reported in prior investigations often required reaction times of 60 to 240 min under standard conditions^77,78^.

The technology used in this study enables the rapid synthesis of the target heterocyclic compounds within 15–25 min at ambient temperature, using Fe3O4 nanoparticles in conjunction with a static magnetic field (SMF) generated by a neodymium magnet. The melting points of the synthesized products aligned with those reported in the literature, thereby validating the successful synthesis of the target compounds.

The significant decrease in reaction time is due to the synergistic interaction between magnetite nanoparticles and the applied magnetic field. The magnetic field may augment molecular alignment and promote interactions between the reactants and the catalytic surface of Fe_3_O_4_ nanoparticles. Furthermore, the catalyst’s magnetic properties may enhance the dispersion and activation of reactants during the reaction.

Consequently, the current magnetic-field-assisted method serves as a straightforward, effective, and eco-friendly alternative to conventional synthetic techniques, offering notable benefits such as reduced reaction times, moderate reaction conditions, and ease of operation.

### Green chemistry assessment and catalyst sustainability

To evaluate the sustainability of the developed synthetic protocol, green chemistry metrics including atom economy (AE), reaction mass efficiency (RME), process mass intensity (PMI), and E-factor were calculated for all synthesized compounds ^79,80^. The synthesized compounds **(1–8)** exhibited significant atom economy (AE) values, reaching up to 98.85% for compound **7**, which highlights the efficiency of the developed protocols in maximizing the incorporation of reactant atoms into the final heterocyclic frameworks. While the isolated yields were moderate (30–34%), the environmental profile of the process remained favorable. The relatively higher PMI and E-factor values are primarily attributed to the bulk use of ethanol as a solvent; however, this is mitigated by its classification as a green bio-based medium ^81,82^, Table 4.

**Table 4:** Green chemistry metrics.

Compound	Yield %	Atom economy %	PMI	E-factor	Remarks
1	30	86.44	25.72	24.72	Atom-efficient MCR process
2	34	90.65	27.48	26.48	High AE due to cyclization
3	30	82.70	35.01	34.01	Efficient condensation
4	34	61.35	37.00	36.00	Facile quinoxaline scaffold build
5	31	55.22	40.69	39.69	Low-waste lactamization route
6	31	66.52	39.93	38.93	Amine formation pathway
7	33	98.85	21.24	30.24	Near-ideal atom economy (99%)
8	31	78.82	43.80	42.80	Direct condensation; stable core

Importantly, the Fe₃O₄ nanocatalyst retained its catalytic efficiency over four successive reaction cycles with negligible loss in activity, confirming its excellent stability under the applied static magnetic field (9000 G). This recyclability significantly reduced both E-factor and PMI values across repeated runs, highlighting the process as a sustainable and environmentally benign synthetic approach.

The use of a static magnetic field in combination with Fe₃O₄ nanoparticles enhanced catalytic efficiency and enabled reactions to be completed within 15–25 min at room temperature, eliminating the need for heating and reducing energy consumption. The Fe₃O₄ nanocatalyst was magnetically separated after each reaction cycle, washed with ethanol, and reused for four consecutive runs without significant loss in catalytic activity, confirming its robustness and recyclability.

## Experimental

### Materials and measurements

All chemicals and reagents were obtained from Aldrich Chemical Co., Inc. (WI, USA). All melting points are uncorrected and were measured using an Electro-thermal IA 9100 apparatus. IR spectra (KBr) were recorded on a Nexus 670 FTIR Nicolet, Fourier transform infrared spectrometer. The 1H and 13C NMR spectra were determined with a JEOL-JNM-LA 500 MHz spectrometer. The chemical shifts are expressed on the δ (ppm) scale using TMS as the standard reference. UV light performed TLC on Merck Silica Gel 60F254 with detection. The permanent neodymium magnet (60Nd) material was sassed by using Vibrating Sample Magnetometer (VSM) measurement methodology in the characterization of permanent magnets. The size of magnetite Fe_3_O_4_ NPs was measured by the transmission electron microscopy imaging technique in the National Research Center in Egypt. X-ray diffraction (XRD) patterns were obtained utilizing a Bruker D8 Discover (Germany) with a copper source (λ = 1.54 Å). The generator parameters were consistently set at 40 kV and 40 mA during the analysis.

## Chemistry

### 1-(6-methyl-4-phenyl-2-thioxo-1,2,3,4-tetrahydropyrimidin-5-yl)ethan-1-one (1)

A mixture of thiourea (0.76 g, 10 mmol, 1.0 equiv), the appropriate aromatic aldehyde (10 mmol, 1.0 equiv), and acetylacetone (1.0 mL, 10 mmol, 1.0 equiv) was dissolved in absolute ethanol (20 mL). Fe₃O₄ nanoparticles (0.23 g, 1 mmol) were then added as a catalyst. The reaction mixture was then exposed to an external magnetic field (9000 Gauss) at room temperature for 15 min. The progress of the reaction was monitored by thin-layer chromatography (TLC) using a methanol/methylene chloride (1:5) solvent system. After completion of the reaction, the resulting precipitate was filtered, washed with 95% ethanol, and dried. The crude product was purified by recrystallization from ethanol to afford compound (1) as a solid in 30% yield, m.p. 183 °C. Blank experiment gave NR after overnight standing at RT without exposure to the magnetic field.

The compound **1** was prepared by optimized reaction conditions and purified by recrystallization in ethanol. White solid 30% yield; mp 183- 185°C; IR (KBr) υ (cm^-1^): 3120(NH), 1720 cm^-1^ (C = O), 1257 cm^-1^ (C = S).^1^H NMR (400 MHz, DMSO-d_6_) δ = 1.2 (s, 3H, CH_3_), 4.21 (s, 3H, CH_3_CO), 7.31 (s, 5H, H_aryl_), 10.60 (s, 1H, NH). Anal Cald. For C_13_H_12_N_2_OS (244.07): C, 63.91; H, 4.95; N, 11.47; S, 13.12. Found C, 63.89; H, 4.93; N, 11.46; S, 13.10.

### General procedure for preparation of compound (2–8)

A mixture of o-phenylenediamine (1.08 g, 10 mmol, 1.0 equiv) and the appropriate carbonyl compound (10 mmol, 1.0 equiv), namely benzaldehyde (1.06 g, 10 mmol, 1.02 mL), acetylacetone (1.00 g, 10 mmol, 1.03 mL), ethyl acetoacetate (1.30 g, 10 mmol, 1.27 mL), diethyl malonate (1.60 g, 10 mmol, 1.51 mL), ethyl 2-cyanoacetate (1.13 g, 10 mmol, 1.07 mL), malononitrile (0.66 g, 10 mmol), or glyoxal (0.58 g, 10 mmol, if pure reagent), was dissolved in absolute ethanol (20 mL). Fe₃O₄ nanoparticles (0.23 g, 1 mmol) were added as a catalyst. The reaction mixture was subjected to an external magnetic field of 9000 Gauss at room temperature for 15 min. In the case of compound 5, the reaction time was extended to 25 min. The progress of the reaction was monitored by thin-layer chromatography (TLC) using methanol/methylene chloride (1:5) as the eluent. After completion, the resulting precipitate was filtered off, washed with 95% ethanol, and dried. The crude products were purified by recrystallization from ethanol to afford the desired compounds (**2–8**) in 30–34% yield. Blank experiments showed no reaction (NR) after overnight standing at room temperature in the absence of a magnetic field.

### 2-phenyl-1H-benzimidazole (2):

Pale yellow solid; yield**:** 34%; m**.**p**.:** 296–297 °C. Molecular formula: C₁₃H₁₀N₂ (M.W. = 194.23). IR (KBr, cm⁻1): 3163 (NH), 3047 (Ar–CH), 1589 (C = N). 1H NMR (400 MHz, DMSO-d₆, δ ppm): 7.21–7.23 (dd, J = 8 Hz, 2H, Ar–H), 7.50–7.63 (m, 5H, Ar–H), 8.19–8.21 (d, J = 8 Hz, 2H, Ar–H), 10.60 (s, 1H, NH). 13C NMR (101 MHz, DMSO-d₆, δ ppm): 151.20, 142.62, 136.92, 135.87, 129.90, 129.28, 126.47, 122.17. Anal. Calcd. for C₁₃H₁₀N₂: C, 80.39; H, 5.19; N, 14.42. Found: C, 80.35; H, 5.17; N, 14.41.

### 2, 4-dimethyl-3 *H* -benzo[ *b* ][1,4]diazepine (3)

Colorless crystals; yield: 30%; m.p.: 130–133 °C. Molecular formula: C₁₁H₁₂N₂ (M.W. = 172.23). IR (KBr, ν cm⁻^1^): 3055 (Ar–CH), 1635 (C = N). ^1^H NMR (400 MHz, DMSO-d₆, δ (ppm)): 2.22 (s, 6H, 2CH₃), 2.64 (s, 2H, CH₂), 6.48–6.58 (dd, J = 8 Hz, 2H, Ar–H), 6.68–6.72 (m, 2H, Ar–H). Anal. Calcd. for C₁₁H₁₂N₂: C, 76.71; H, 7.02; N, 16.27. Found: C, 76.69; H, 7.00; N, 16.25.

### 3-methyl-1,2-dihydroquinoxaline (4)

Faint yellow solid; yield: 34%; m.p.: 237–240 °C. Molecular formula: C₉H₁₀N₂ (M.W. = 146.19). IR (KBr, ν cm⁻^1^): 3174 (N–H), 3097 (Ar–C–H), 2873 (aliphatic C–H), 1624 (C = N). ^1^H NMR (400 MHz, DMSO-d₆, δ ppm): 2.49 (s, 3H, CH₃), 4.08 (s, 2H, CH₂), 7.10–7.12 (dd, J = 8 Hz, 2H, Ar–H), 7.44–7.47 (dd, J = 8 Hz, 2H, Ar–H), 8.15 (br s, 1H, NH). Anal. Calcd. for C₉H₁₀N₂: C, 73.94; H, 6.89; N, 19.16. Found: C, 73.92; H, 6.87; N, 19.10.

### 3, 4-dihydroquinoxalin-2(1H)-one (5)

Beige solid; Yield: 31%; mp 144–147 °C. IR (KBr, cm⁻^1^): 3173–3178 (2NH), 3059 (Ar–H), 1665 (C = O), 1581 (C = C).^1^H NMR (400 MHz, DMSO-d₆, δ ppm): 4.09 (s, 2H, CH₂), 6.31–6.51 (d, 1H, J = 8 Hz, Ar–H), 6.92–6.94 (d, J = 8 Hz, 1H, Ar–H), 7.46 (s, 1H, NH), 7.46–7.56 (m, 1H, Ar–H), 7.89–7.91 (m, 1H, Ar–H), 10.40 (s, 1H, NHCO). ^13^C NMR (101 MHz, DMSO-d₆, δ ppm): 45.53, 102.44, 114.63, 121.81, 126.95, 128.16, 140.50, 166.27. Anal. Calcd for C₈H₈N₂O (148.16): C, 64.85; H, 5.44; N, 18.91%. Found C, 64.82; H, 5.42; N, 18.89%.

### 3, 4-dihydroquinoxalin-2-amine (6)

Faint yellow solid; Yield: 31%; mp 145–147 °C. IR (KBr, cm⁻^1^): 3312–3305 (NH_2_), 3178 (NH), 3059 (Ar–H), 1639 (C = N). ^1^H NMR (400 MHz, DMSO-d₆, δ ppm): 3.87 (s, 2H, CH₂), 6.45 (s, 2H, NH₂), 6.92–6.94 (d, J = 8 Hz, 2H, Ar–H), 7.22 (s, 1H, NH), 7.57–7.59 (m, 1H, Ar–H), 7.90–7.92 (m, 1H, Ar–H). Anal. Calcd for C₈H₉N₃ (147.18): C, 65.29; H, 6.16; N, 28.55%. Found: C, 65.27; H, 6.14; N, 28.53%.

### 3-amino-1,2-dihydroquinoxaline-2-carbonitrile (7)

Brown solid; Yield: 33%; m.p. 184–186 °C. Molecular formula: C₉H₈N₄. IR (KBr, ν, cm⁻^1^): 3332 (NH₂), 3244 (N–H), 2194 (C≡N), 1651 (C = N).^1^H NMR (400 MHz, DMSO-d₆, δ ppm): 4.39 (s, 1H, CH), 6.37–6.39 (dd, J = 8 Hz, 2H, Ar–H), 6.49–6.52 (m, 2H, Ar–H), 7.55 (s, 1H, NH₂), 12.58 (s, 1H, NH). ^13^C NMR (101 MHz, DMSO-d₆, δ ppm): 53.90, 115.15, 117.08, 117.98, 122.23, 122.50, 135.26, 145.30, 156.92. Anal. Calcd. for C₉H₈N₄ (172.19): C, 62.78; H, 4.68; N, 32.54. Found: C, 62.76; H, 4.67; N, 32.52.

### Quinoxaline (8)

White semi-solid; molecular formula: C₈H₆N₂; yield: 31%. IR (KBr, ν, cm⁻^1^): 1500 (C = C). ^1^H NMR (400 MHz, DMSO-d₆, δ ppm): 7.88–7.90 (dd, J = 8 Hz, 2H, Ar–H), 8.11–8.14 (m, 2H, Ar–H), 8.97 (s, 2H, Ar–H). Elemental analysis: Calcd for C₈H₆N₂ (M.W. 130.15): C, 73.83; H, 4.65; N, 21.52%. Found: C, 73.81; H, 4.63; N, 21.50%.

### Catalyst recycling and reusability

After the reaction was finished, the catalyst was magnetically separated by inserting a neodymium magnet (20 × 3 × 50 mm) into the reaction jar. Before being employed again in subsequent cycles, the recovered catalyst was thoroughly cleaned with absolute ethanol and then vacuum-dried. Four successive reaction runs under ideal circumstances were used to assess the catalyst’s recyclability.

## Conclusion

This work introduces an innovative and sustainable synthetic method that leverages the synergistic interplay between Fe₃O₄ nanoparticles and a neodymium-induced static magnetic field to effectively synthesize physiologically relevant heterocyclic compounds. The results affirm the critical role of the magnetic field in facilitating the reactions, as no conversion occurred in its absence, underscoring a distinct magnetic-field-assisted activation mechanism. This method facilitates the synthesis of pyrimidine, benzimidazole, quinoxaline, and benzodiazepine derivatives at ambient temperatures, resulting in shorter reaction times and straightforward recovery of the magnetic catalyst, thereby providing significant benefits in energy efficiency, environmental sustainability, and operational ease. Subsequent research will focus on improving reaction efficiency, expanding substrate scope, elucidating the mechanism of magnetic-field activation, and exploring the application of this approach to other significant organic transformations.

## Supplementary Information

Supplementary Information.

## Data Availability

All data generated or analyzed during this study are included in this published article [and its supplementary information files].

## Abbreviations

SMF: Static magnetic field
TEM: Transmission electron microscopy
VSM: Vibrating sample magnetometer
NPs: Nanoparticles
