# Supplementary material for: Neodymium magnetic field meets nanocatalysis: a sustainable route to novel azines and condensed heterocycles
Source: Sci Rep. 2026 May 21;16:15859. doi: 10.1038/s41598-026-51258-8 (PMC13194876; doi:10.1038/s41598-026-51258-8)
Supplement: Supplementary file 1 — Supplementary Information. [file 41598_2026_51258_MOESM1_ESM.pdf]

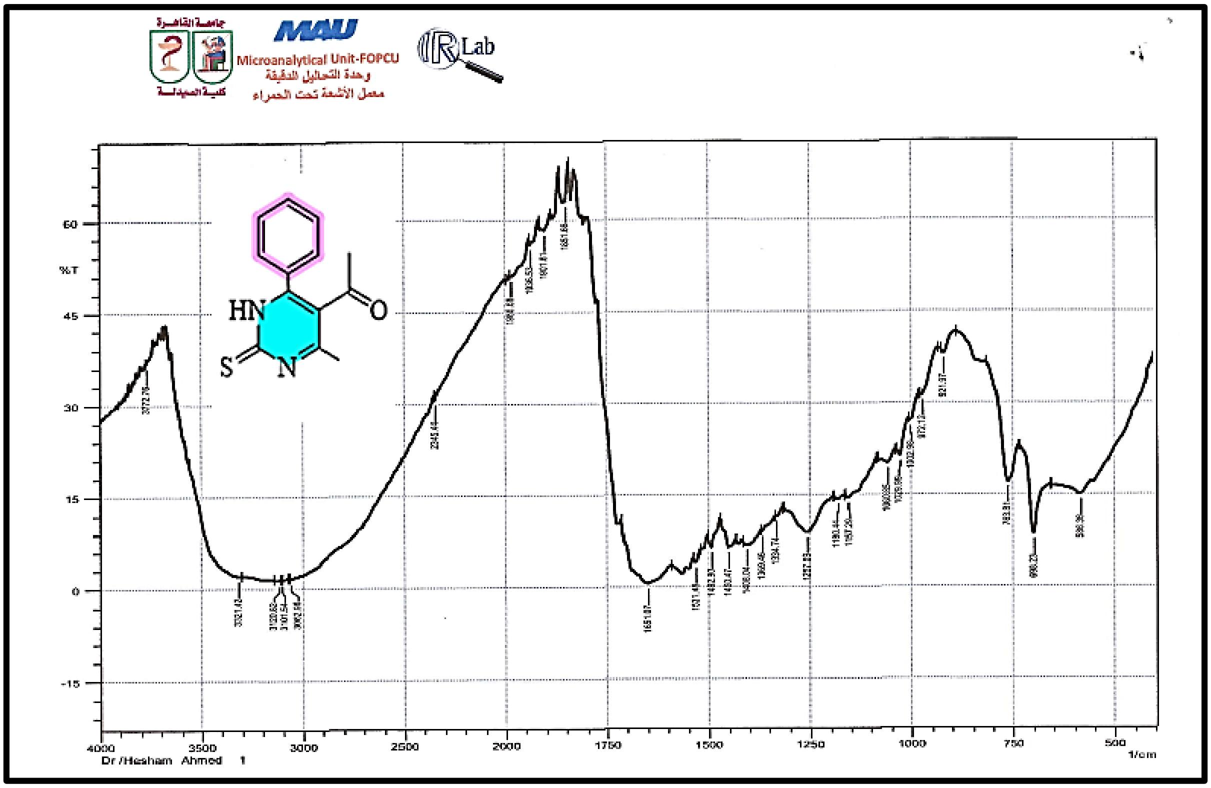

IR Spectrum of compound 1

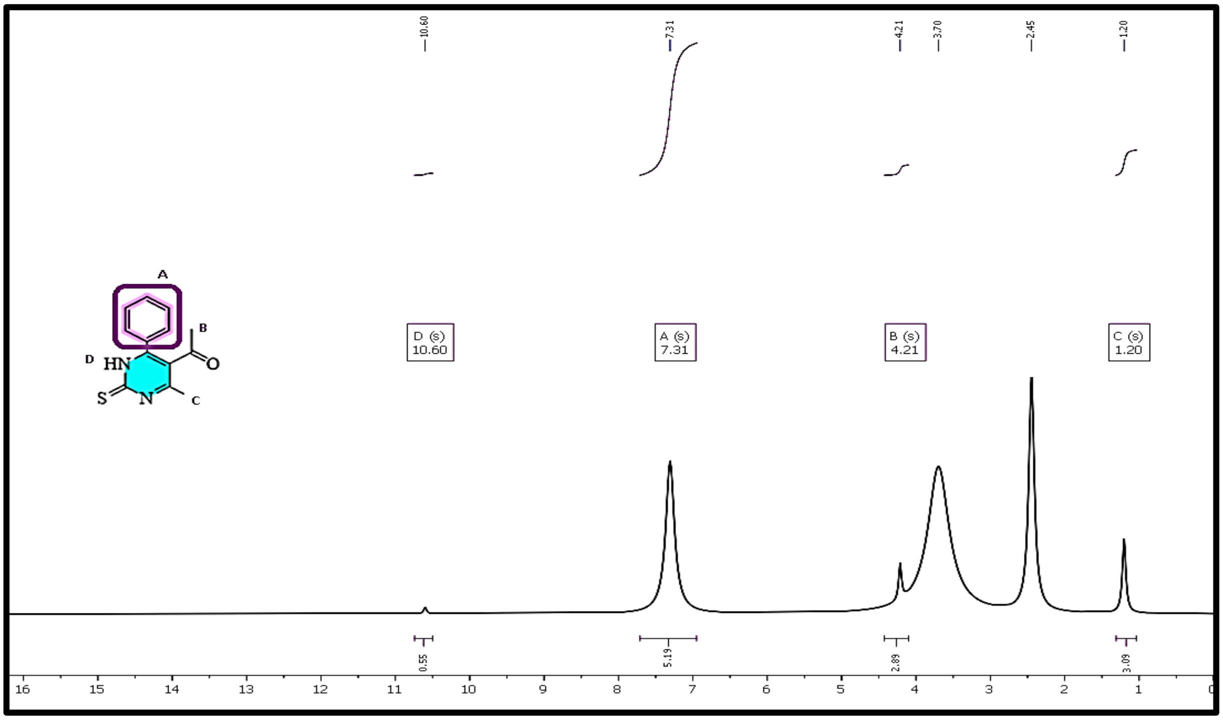

^{1}H-NMR Spectrum of compound 1 by DMSO-d_6

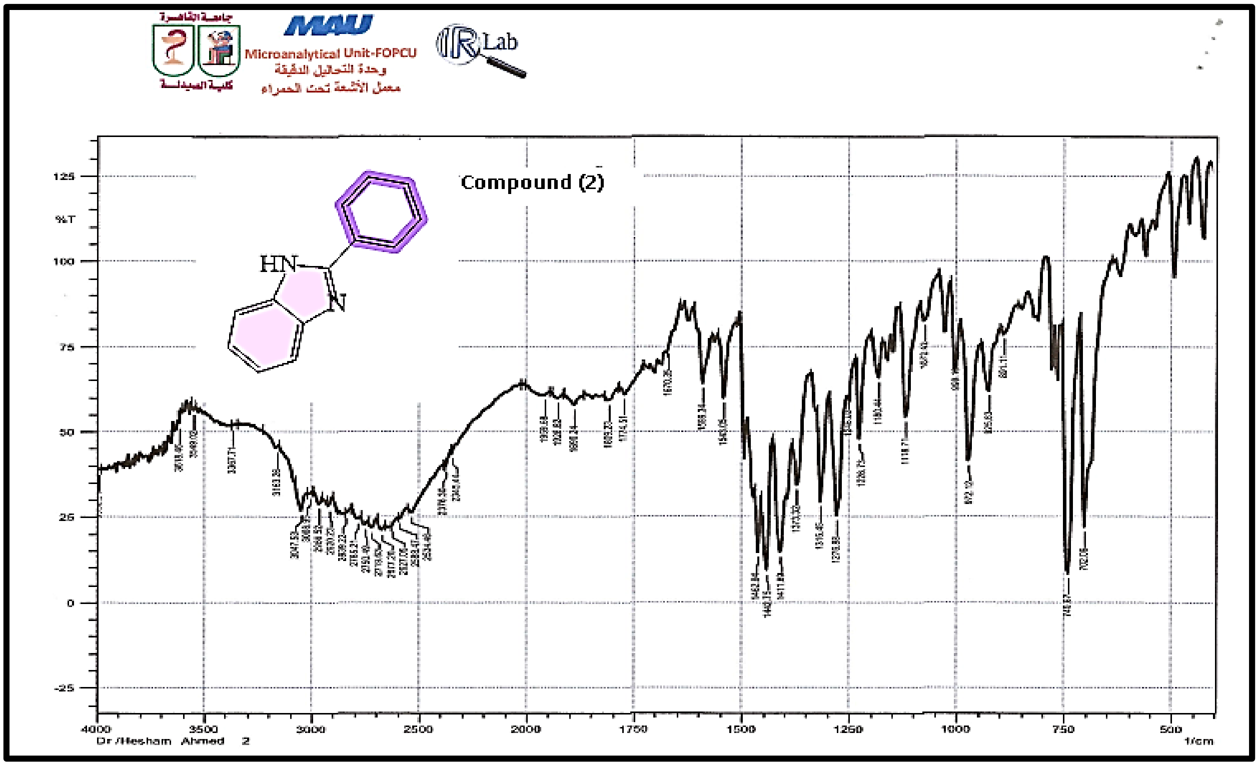

IR Spectrum of compound 2

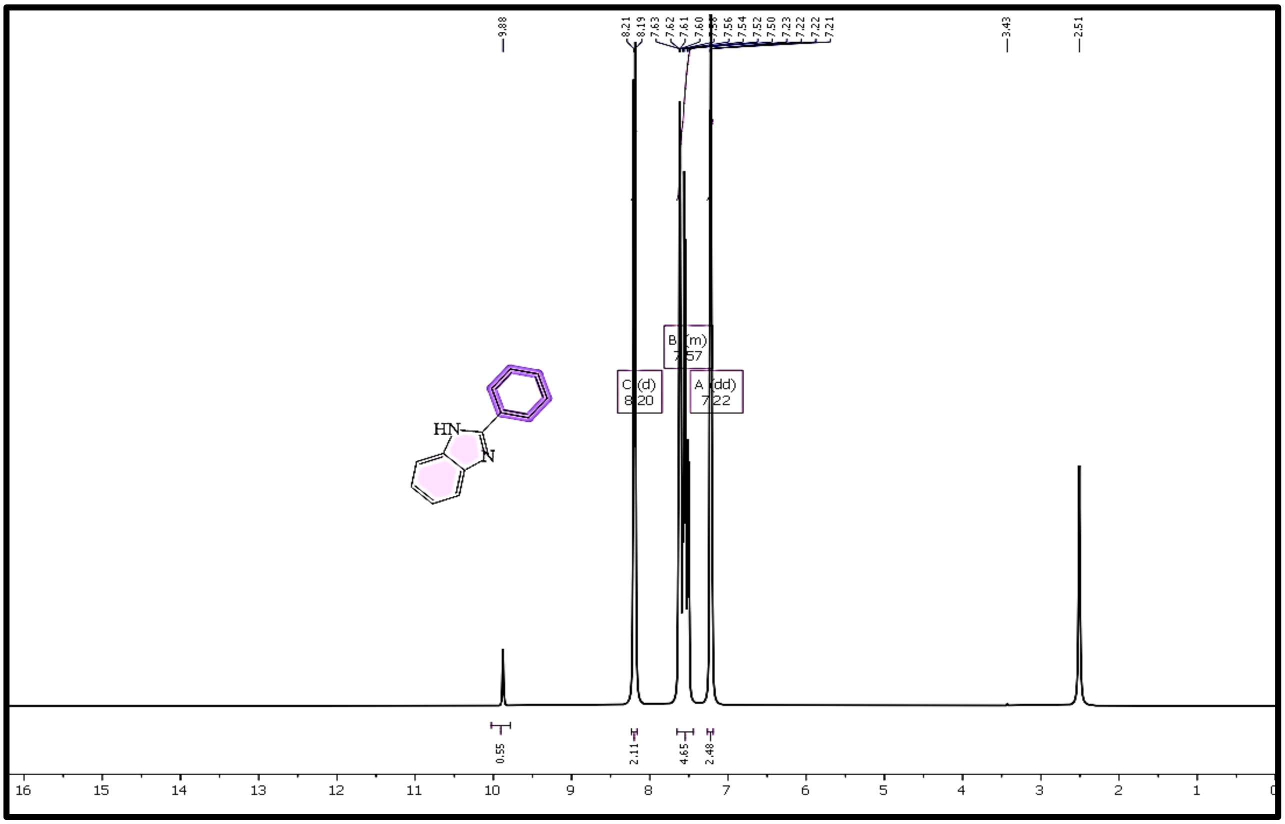

^{1}H-NMR Spectrum of compound 2 by DMSO-d_6

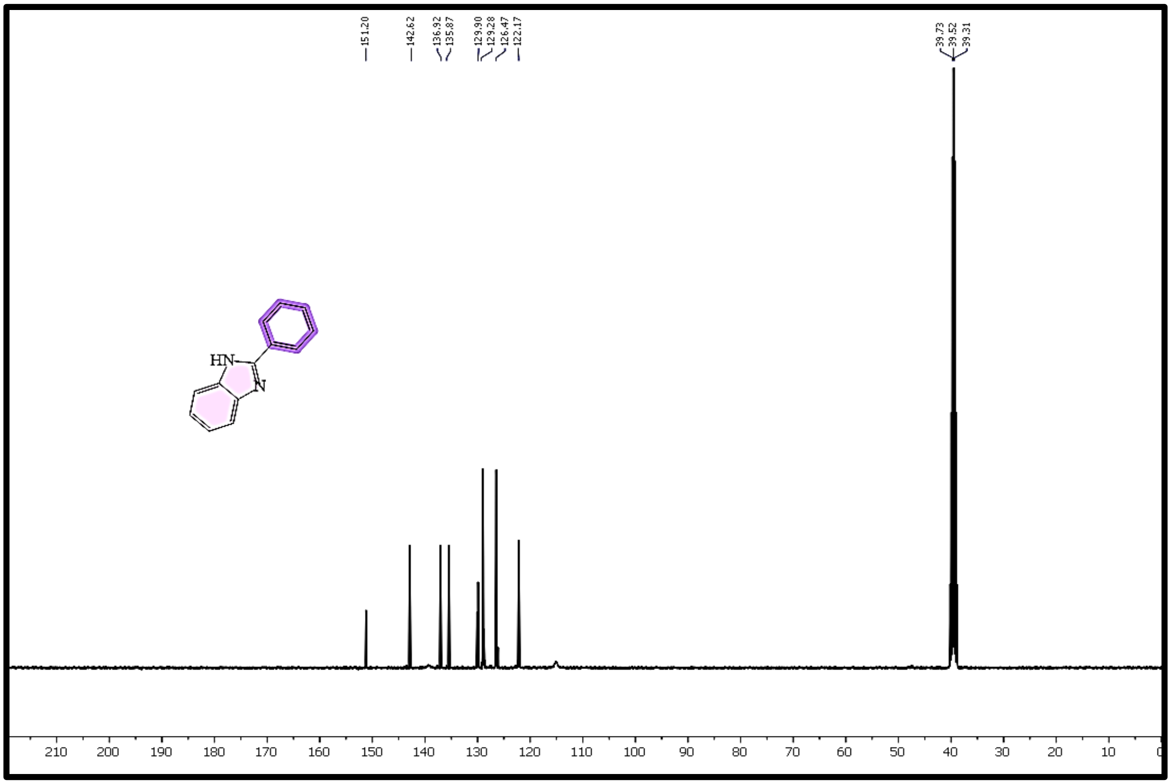

^{13}C-NMR Spectrum of compound 2 by DMSO-*d_6*

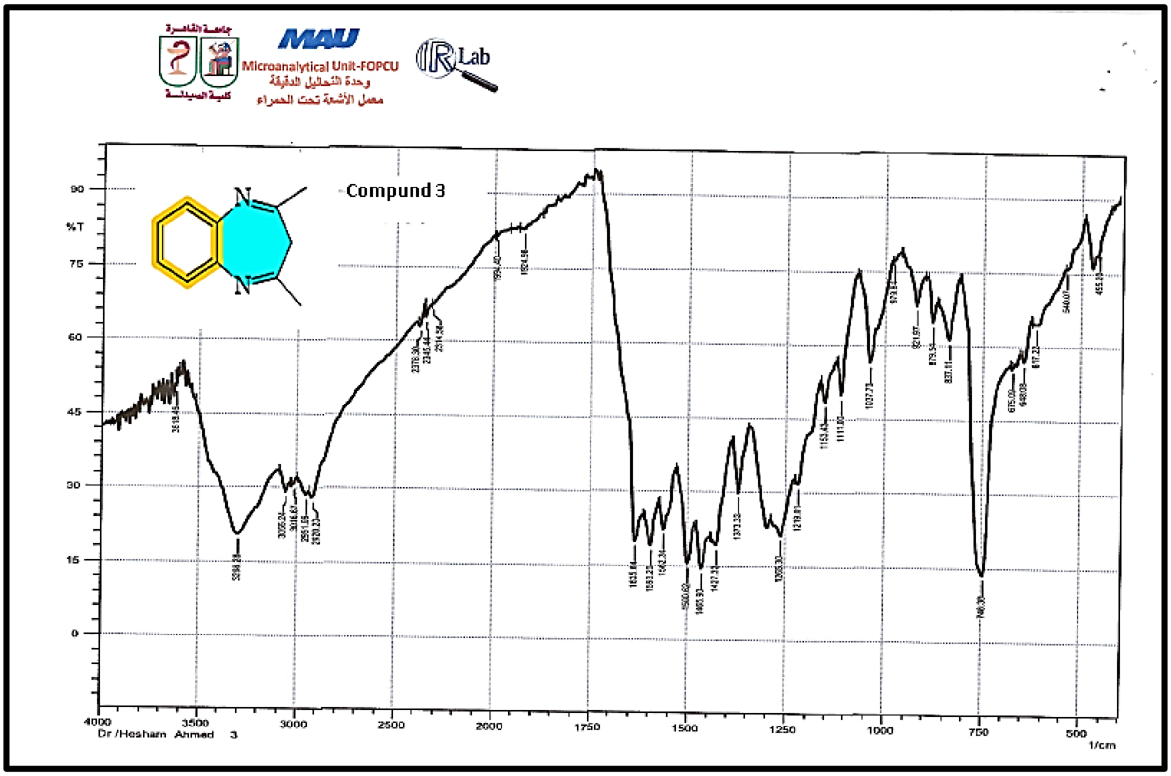

IR Spectrum of compound 3

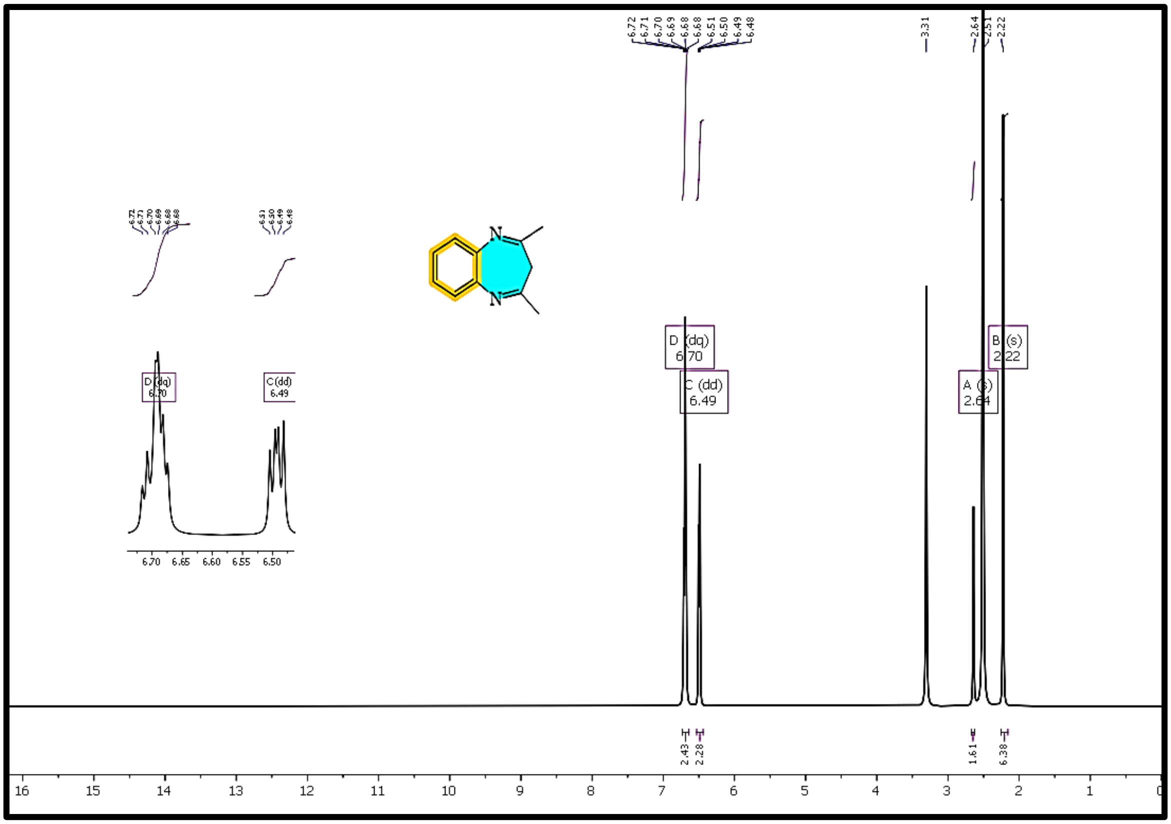

^{1}H-NMR Spectrum of compound 3 by DMSO-d_6

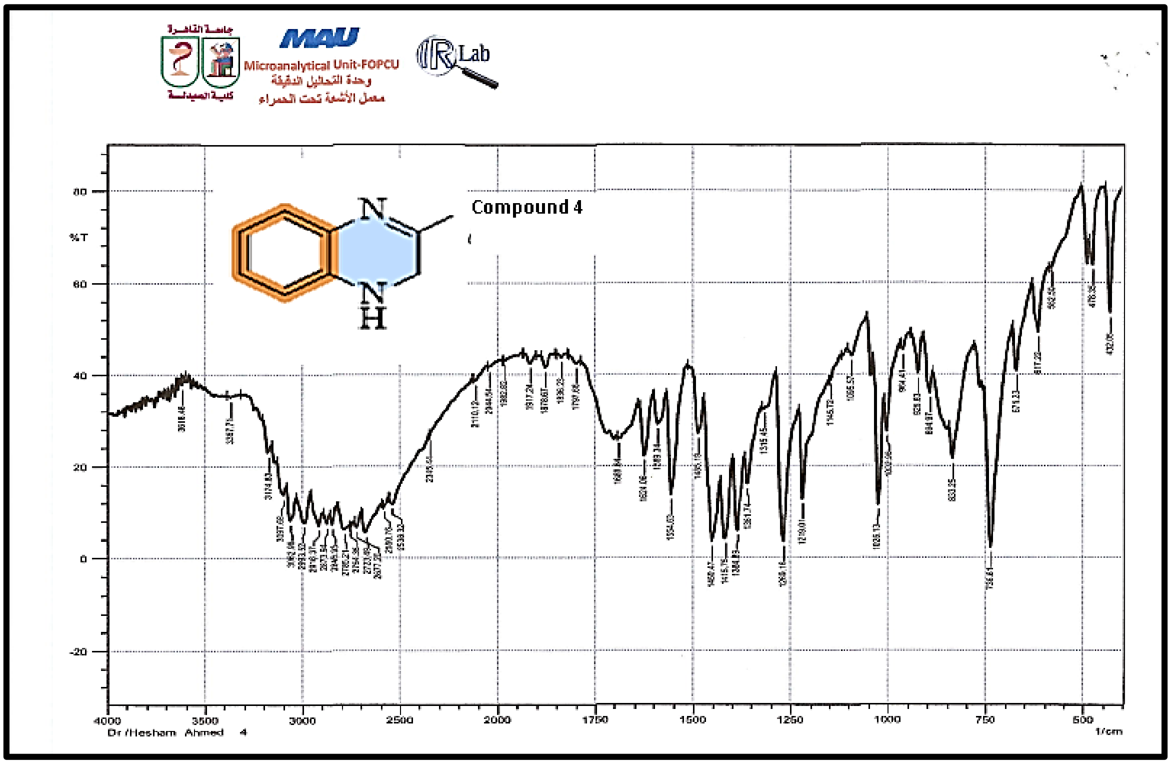

IR Spectrum of compound 4

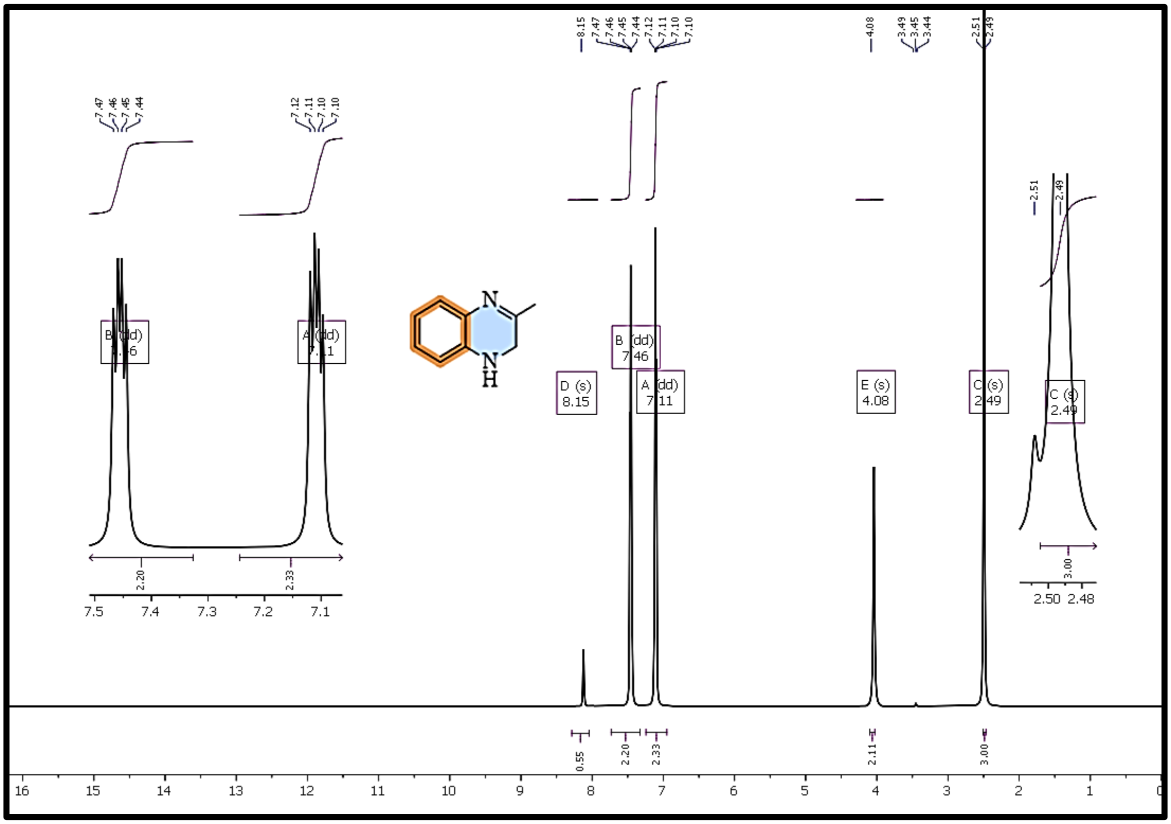

^{1}H-NMR Spectrum of compound 4 by DMSO-d_6

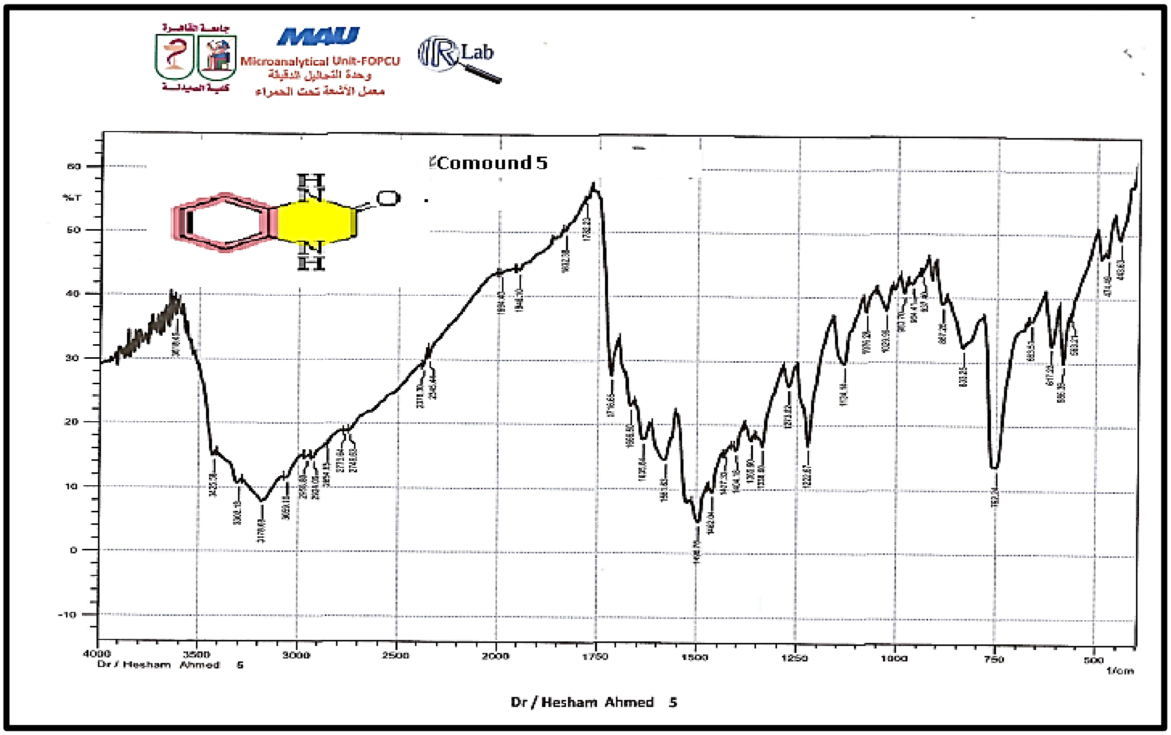

IR Spectrum of compound 5

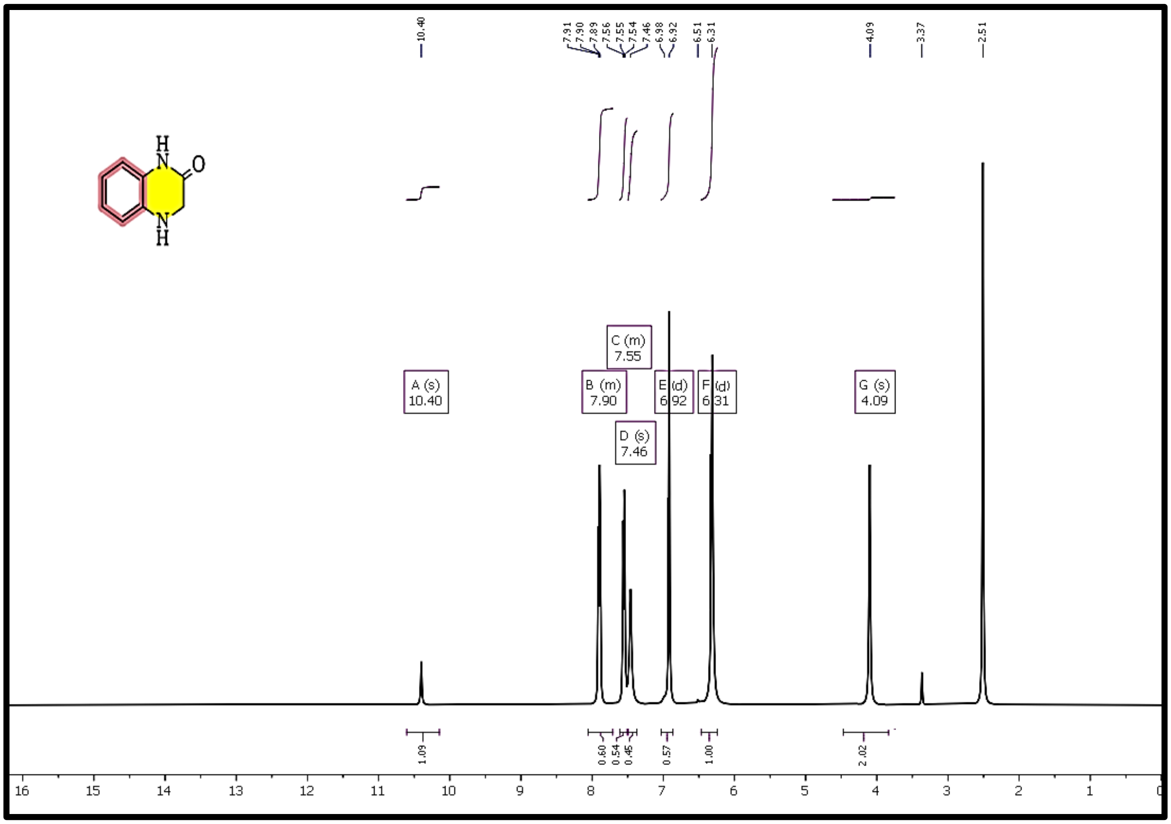

^{1}H-NMR Spectrum of compound 5 by DMSO-d_6

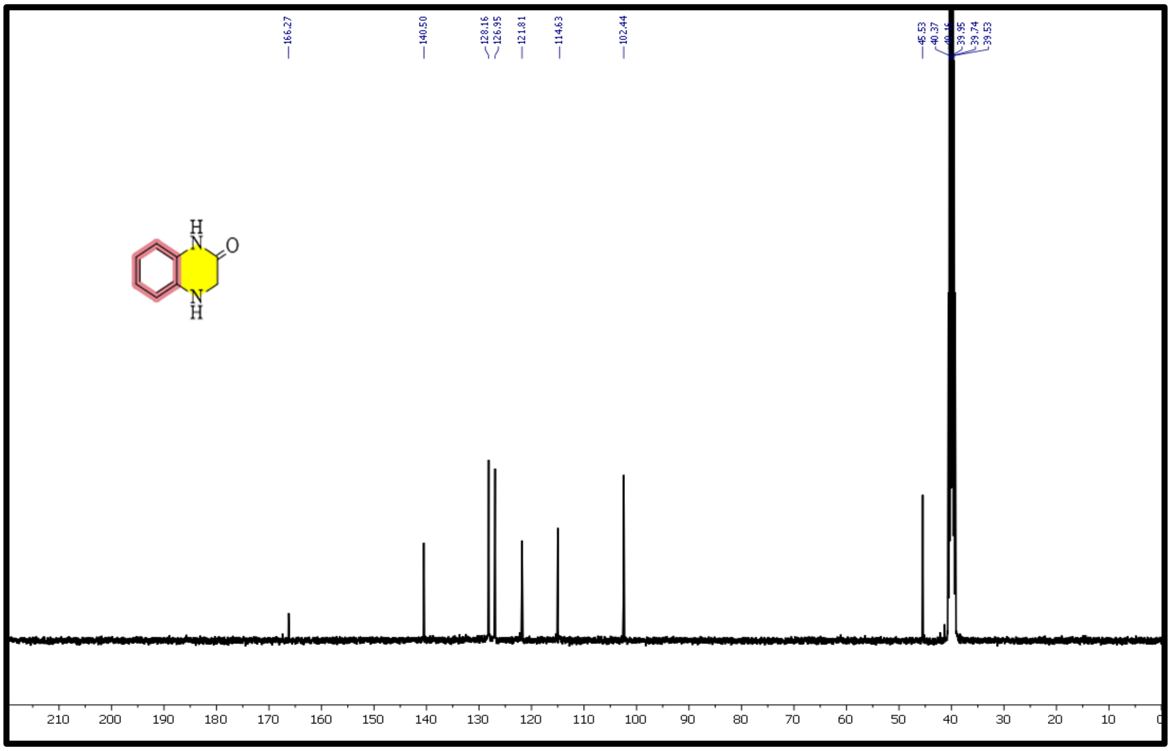

^{13}C-NMR Spectrum of compound 5 by DMSO-d_6

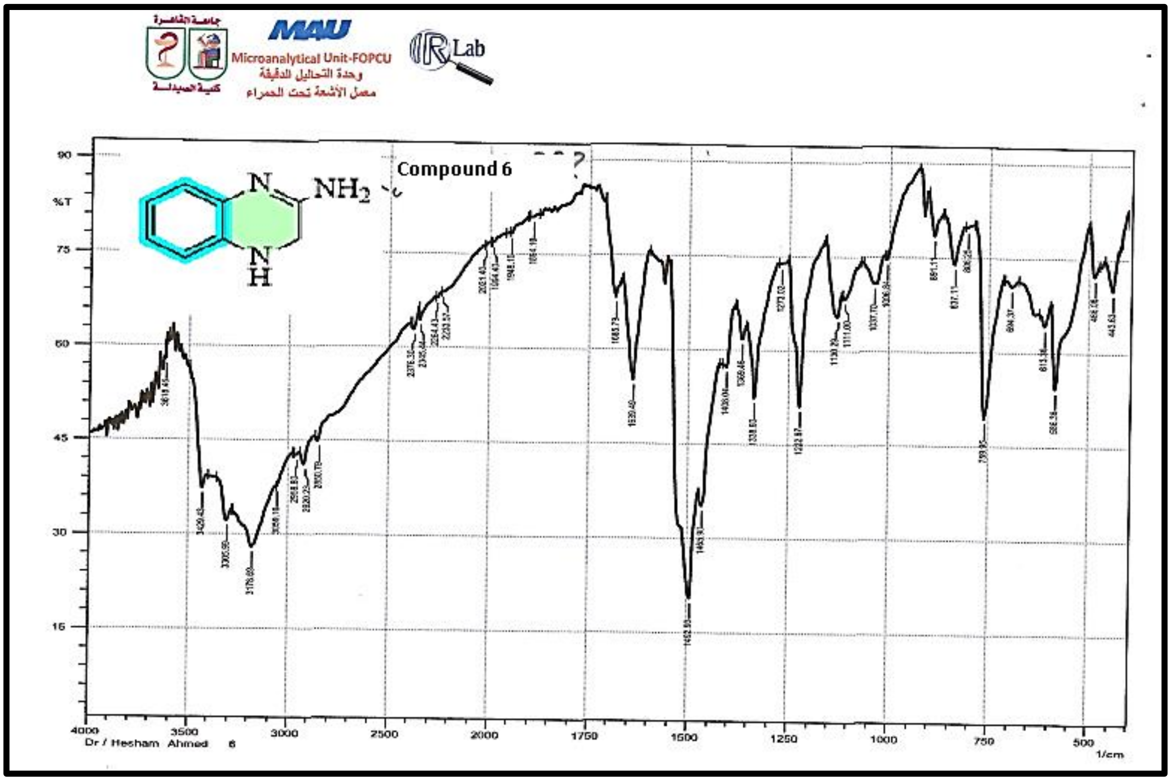

IR Spectrum of compound 6

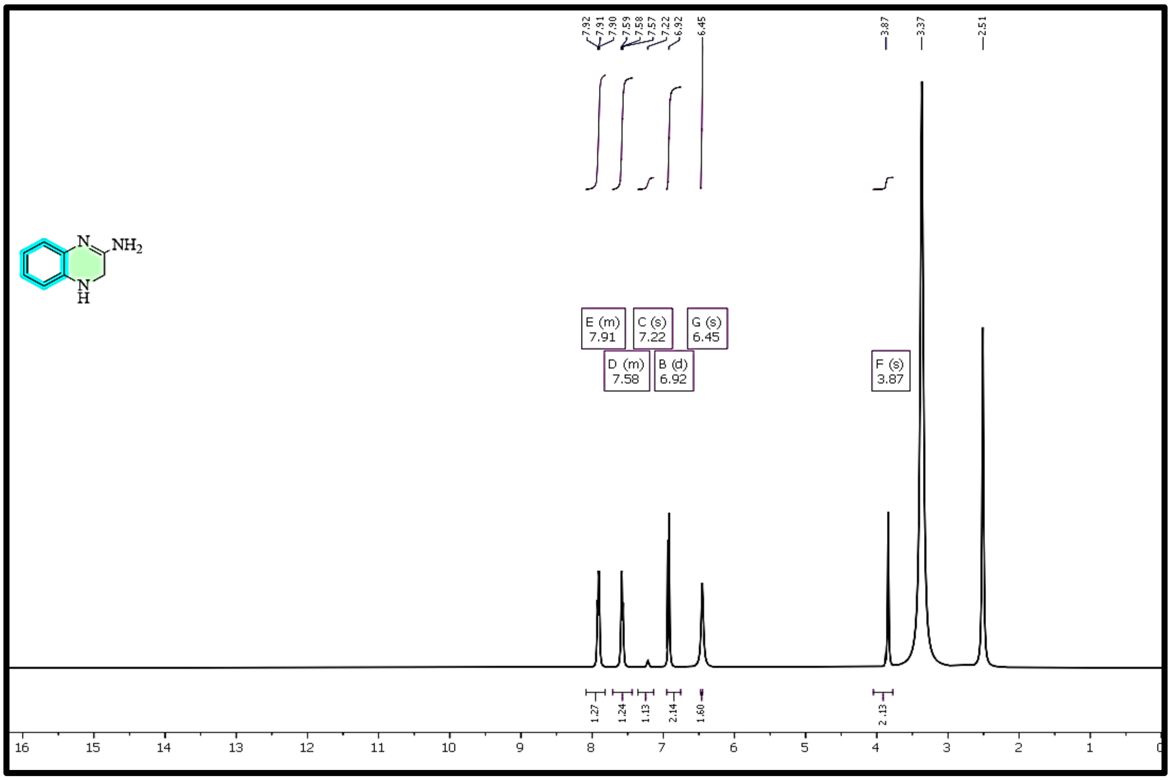

^{1}H-NMR Spectrum of compound 6 by DMSO-d_6

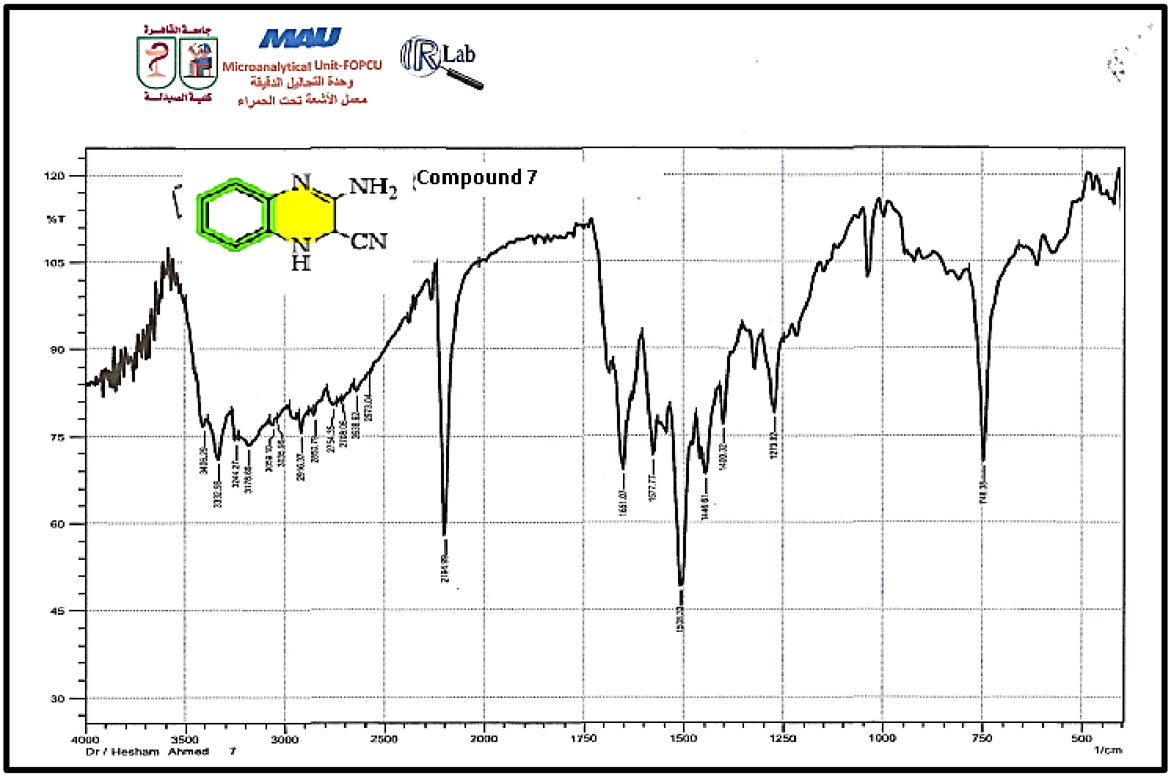

IR Spectrum of compound 7

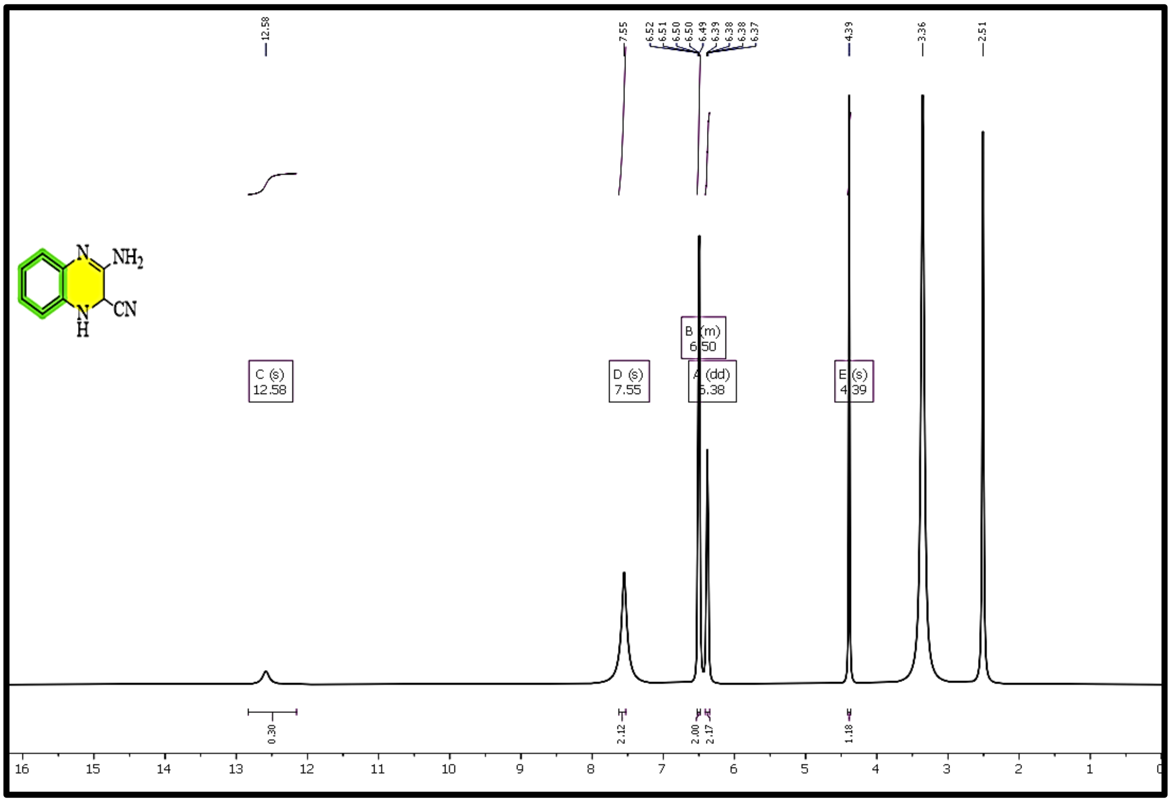

^{1}H-NMR Spectrum of compound 7 by DMSO-d_6

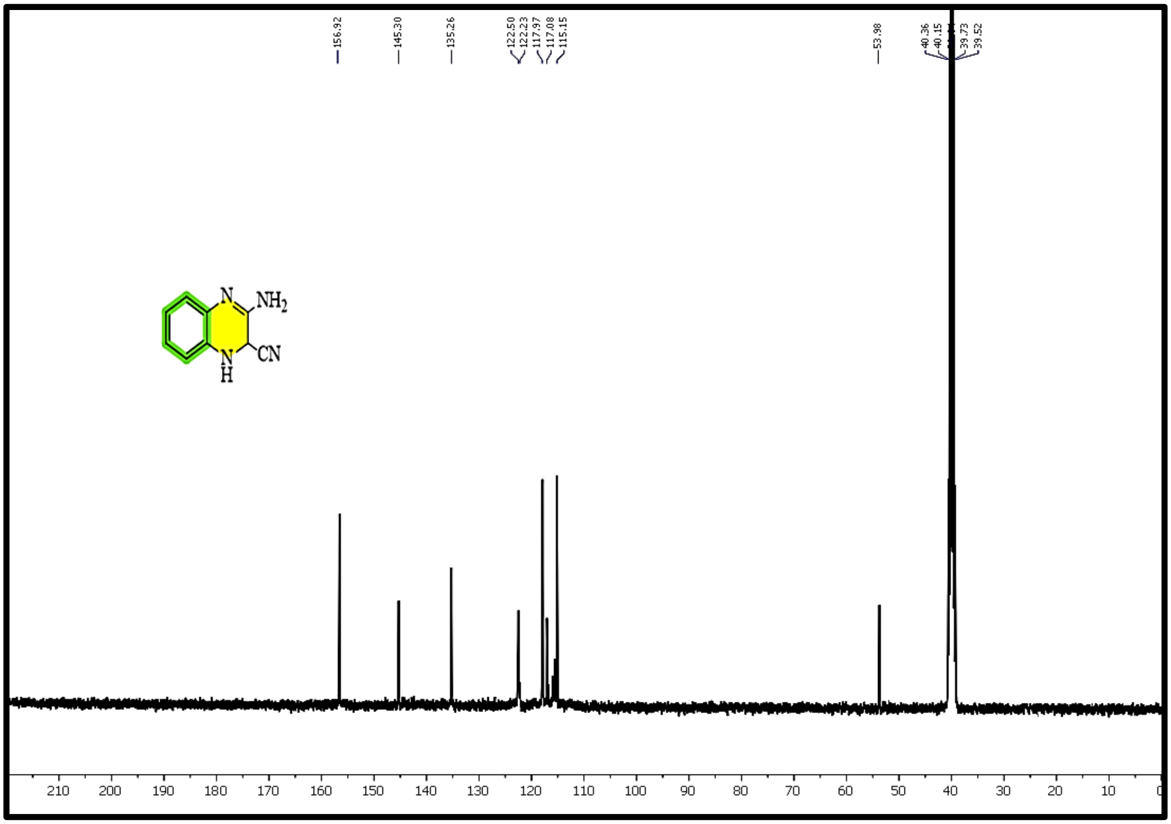

^{13}C-NMR Spectrum of compound 7 by DMSO-d_6

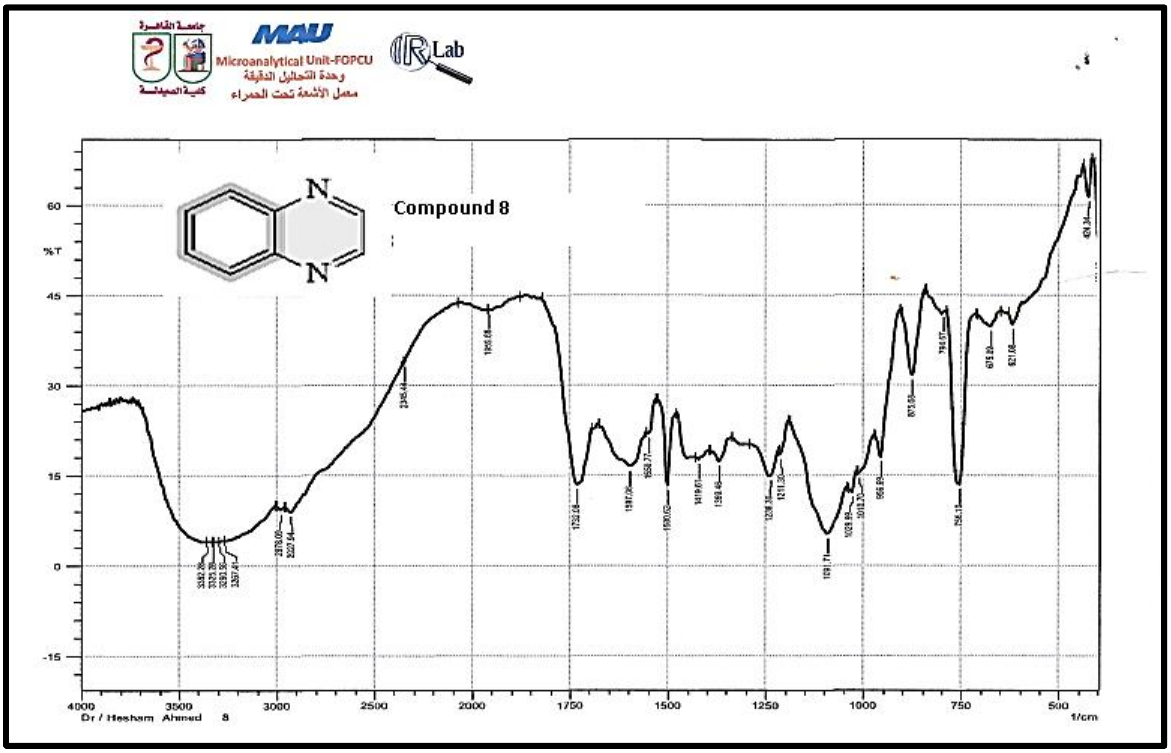

IR Spectrum of compound 8

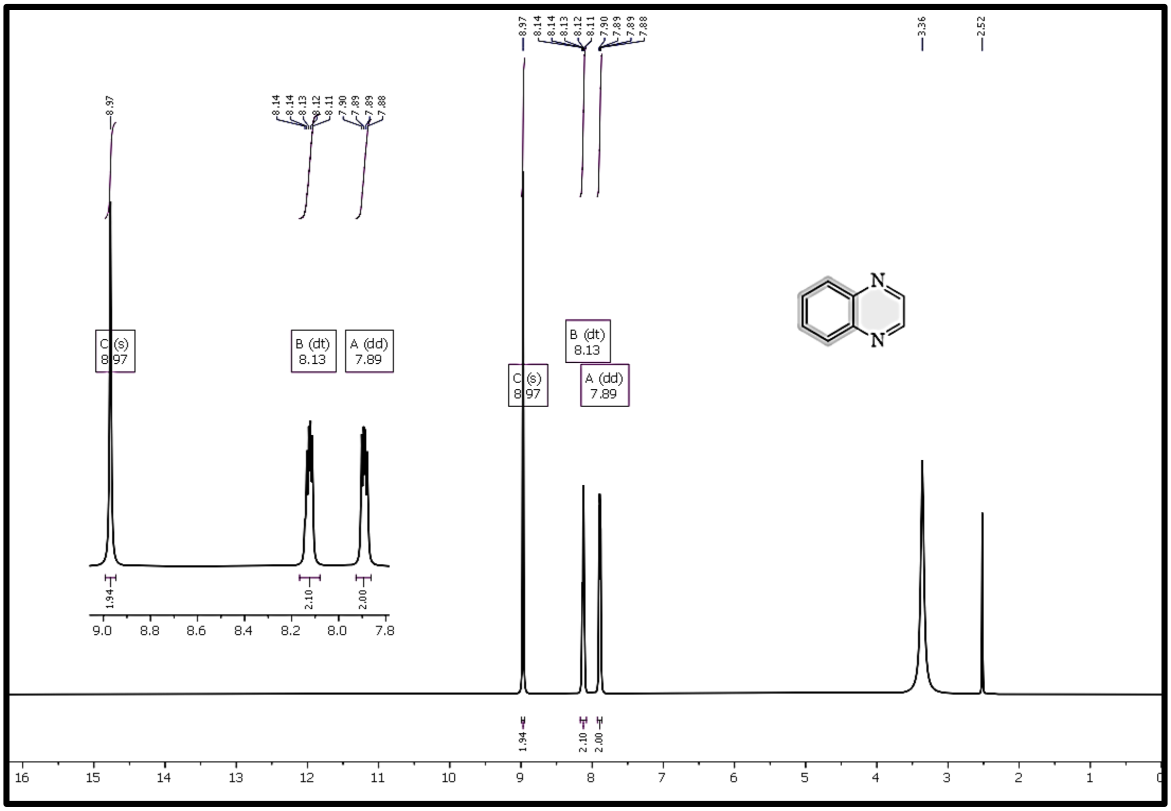

^{1}H-NMR Spectrum of compound 8 by DMSO-d_6
